# AMPK activation protects against prostate cancer by inducing a catabolic cellular state

**DOI:** 10.1016/j.celrep.2023.112396

**Published:** 2023-04-15

**Authors:** Lucy Penfold, Angela Woods, Alice E. Pollard, Julia Arizanova, Eneko Pascual-Navarro, Phillip J. Muckett, Marian H. Dore, Alex Montoya, Chad Whilding, Louise Fets, Joao Mokochinski, Theodora A. Constantin, Anabel Varela-Carver, Damien A. Leach, Charlotte L. Bevan, Alexander Yu. Nikitin, Zoe Hall, David Carling

**Affiliations:** 1 MRC London Institute of Medical Sciences, Hammersmith Hospital Campus, Imperial College London, London W12 0NN, UK; 2 Institute of Clinical Sciences, Imperial College London, London, UK; 3 Imperial Centre for Translational and Experimental Medicine, Department of Surgery and Cancer, Imperial College London, Hammersmith Hospital Campus, London, UK; 4 Department of Biomedical Sciences and Cornell Stem Cell Program, Cornell University, Ithaca, NY, USA; 5 Biomolecular Medicine, Division of Systems Medicine, Department of Metabolism, Digestion, and Reproduction, Imperial College London, London, UK; 6 Lead contact

## Abstract

Emerging evidence indicates that metabolic dysregulation drives prostate cancer (PCa) progression and metastasis. AMP-activated protein kinase (AMPK) is a master regulator of metabolism, although its role in PCa remains unclear. Here, we show that genetic and pharmacological activation of AMPK provides a protective effect on PCa progression *in vivo*. We show that AMPK activation induces PGC1α expression, leading to catabolic metabolic reprogramming of PCa cells. This catabolic state is characterized by increased mitochondrial gene expression, increased fatty acid oxidation, decreased lipogenic potential, decreased cell proliferation, and decreased cell invasiveness. Together, these changes inhibit PCa disease progression. Additionally, we identify a gene network involved in cell cycle regulation that is inhibited by AMPK activation. Strikingly, we show a correlation between this gene network and *PGC1α* gene expression in human PCa. Taken together, our findings support the use of AMPK activators for clinical treatment of PCa to improve patient outcome.

## In brief

Penfold et al. show that AMPK activation protects against prostate cancer progression *in vivo*. AMPK activation switches prostate cancer cells to a tumor-suppressive catabolic state, increasing PGC1α expression and mitochondrial biogenesis while inhibiting lipogenic potential. In parallel, AMPK inhibits a cell cycle gene network associated with prostate cancer progression.

## Graphical Abstract

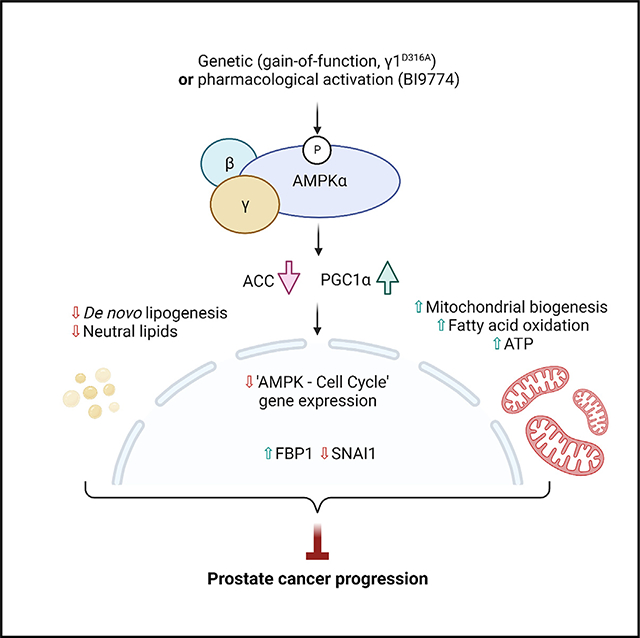

## INTRODUCTION

Prostate cancer (PCa) is now the most common cancer in men in the United Kingdom and United States and second leading cause of cancer-related death.^1^ Advanced PCa is invariably fatal because of acquired resistance to current therapies, highlighting an unmet need for new therapies to better treat this disease.^2,3^

Cellular transformation and cancer progression are reliant on metabolic dysregulation.^4^ Previous studies have shown that increased lipogenesis promotes PCa progression, including metastatic disease, in mice lacking the tumor suppressor phosphatase and tensin homolog (PTEN).^4,5^ AMP-activated protein kinase (AMPK) is a master regulator of energy metabolism, inhibiting anabolic pathways, such as fatty acid synthesis, and promoting catabolic pathways, such as fatty acid oxidation, to maintain cellular ATP levels.^6^ While there are conflicting reports regarding the role of AMPK in PCa, our recent studies support a tumor-suppressive role for AMPK activation. Previously, we have shown that deletion of *Ampkβ1* resulted in earlier onset of adenocarcinoma in a pre-clinical mouse model that lacks expression of *Pten* in the prostate.^7^

PTEN is a negative regulator of cell growth and transformation via downregulation of phosphatidylinositol 3-kinase (PI3K) signaling. Genomic aberrations of *PTEN* are among the most common in human PCa; *PTEN* is progressively lost through disease progression, and complete loss is predominant in advanced and metastatic disease.^8^The prostate-specific *Pten*-null PCa mouse is an important and clinically relevant model that accurately recapitulates the different stages of the human disease, albeit on a more rapid timescale.^9^

Here, we report that genetic activation of AMPK in the *Pten*-null PCa mouse model has a dramatic protective effect, inhibiting disease progression. Furthermore, we find that pharmacological activation of AMPK using a potent and specific direct AMPK activator, BI9774,^10^ reduces tumor growth in a xenograft model of castration-resistant PCa (CRPC). We find that AMPK activation leads to increased expression of peroxisome proliferator-activated receptor gamma co-activator 1 alpha (PGC1α), inducing a catabolic metabolic program that suppresses tumor growth. In addition, AMPK activation leads to a reduction in a transcriptional network of 32 genes negatively correlated with disease-free survival in human PCa that are all involved in regulation of the cell cycle. Of note, this gene network was found to be inversely correlated with PGC1α gene expression in PCa patients. These findings provide the basis for the tumor-suppressive effect of AMPK and strongly support use of AMPK activators for treatment of PCa.

## RESULTS

### Genetic AMPK activation protects against PCa disease progression *in vivo*

To examine the effect of AMPK activation on PCa, we made use of a conditional genetic gain-of-function AMPK mouse model harboring a mutation of aspartic acid residue 316 to alanine on the γ1 subunit.^11,12^ We crossed mice with prostate-specific deletion of *Pten* with mice expressing *D316A* γ*1* specifically in prostate epithelial cells, using probasin-cre^13^ to generate *Pten*^−/−^ and *Pten*^−/−^;*Ampk*^ACT^. As an additional control for insertion of the D316A γ1 transgene, we used mice expressing wild-type (WT) γ1 (*Pten*^−/−^;*Ampk*^*WT*^). In previous studies using this model, we did not observe any overt phenotype in control mice with global or tissue-specific expression of WT γ1 because these mice showed no change in AMPK expression or activity relative to non-transgenic mice.^11,12,14^ Consistent with our previous studies, we found no differences in PCa disease progression between *Pten*^−/−^ and *Pten*^−/−^;*Ampk*^WT^ mice (Figure S1A), and we therefore combined *Pten*^−/−^ and *Pten*^−/−^;*Ampk*^WT^ mice to generate a single control group, referred to as *Pten*^−/−^ mice. We also confirmed that expression of the γ1 transgene had no obvious effect on the levels of endogenous α and β subunit proteins and that the transgene was expressed in anterior and dorsolateral ventral prostate lobes (Figure S1B). To confirm AMPK activation following expression of the D316A γ1 transgene, we generated organoid cultures from prostate epithelial cells isolated from WT, *Pten*^−/−^, and *Pten*^−/−^;*Ampk*^ACT^ mice. Phosphorylation of serine 473 on AKT (also known as protein kinase B) was increased in organoids isolated from either *Pten*^−/−^ or *Pten*^−/−^;*Ampk*^ACT^ mice compared with WT mice (Figure S1C). Expression of the γ1 transgene (C-terminally FLAG tagged) was confirmed along with AMPK activation based on levels of acetylcoenzyme A (CoA) carboxylase (ACC) phosphorylation (Figure S1C). Phosphorylation of ACC was found to be increased by 2- to 3-fold in organoids derived from *Pten*^−/−^;*Ampk*^ACT^ mice compared with *Pten*^−/−^ mice, consistent with our previous studies in liver.^12^ In mice with WT Pten expression, AMPK activation had no effect on prostate weight at ~24 weeks of age or following high-fat diet (HFD) feeding for 16 weeks (from 8–24 weeks of age; Figure S1D). Furthermore, male mice expressing the D316A γ1 transgene were fertile, indicating normal prostate function.

**Figure 1.**
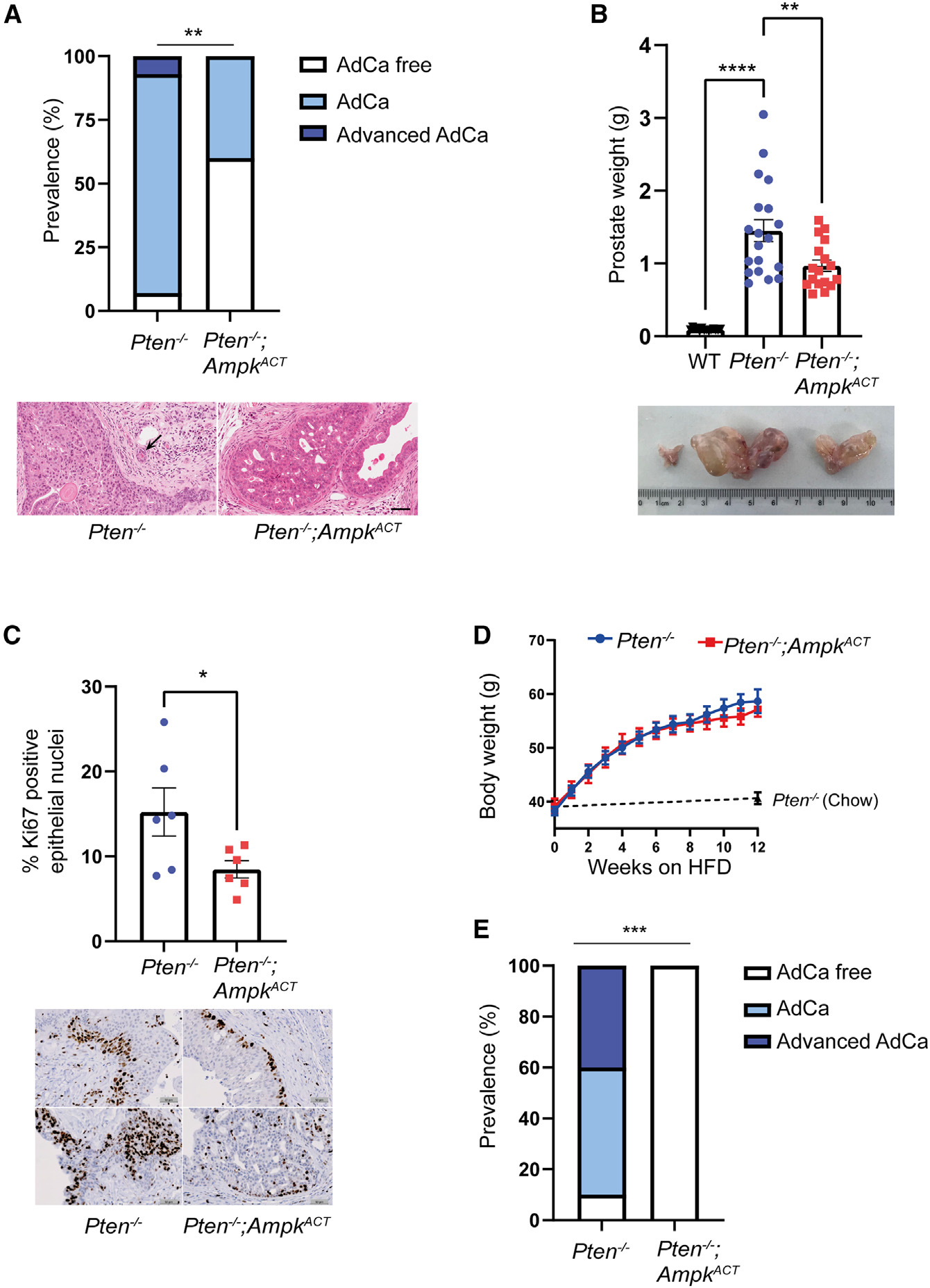
AMPK activation protects against disease progression in a *Pten*^−/−^ mouse model of prostate cancer (A) Quantification of pathological grading from H&E-stained sections isolated from the dorsolateral prostate of 1-year-old mice. AdCa, adenocarcinoma. Significant difference between the AdCa-free group vs. AdCa and advanced AdCa is shown using Fisher’s exact test (two-sided p value); **p < 0.01 (n = 15 for *Pten*^−/−^, n = 10 for *Pten*^−/−^;*Ampk*^ACT^). A representative H&E-stained prostate section isolated from either a *Pten*^−/−^ or *Pten*^−/−^;*Ampk*^ACT^ mouse is shown below and illustrates AdCa or PIN4, respectively. The area of stromal invasion by AdCa in the section from the *Pten*^−/−^ mouse is indicated by an arrow. Scale bar, 60 μm. (B) Weight of whole prostate isolated from 1-year-old mice. A representative image of prostates from wild-type (WT), *Pten*^−/−^, and *Pten*^−/−^;*Ampk*^ACT^ mice is shown. Data are means ± SEM (n = 14–19 per genotype). One-way ANOVA with Tukey’s post hoc test was used to determine difference between genotypes; **p < 0.01, ****p < 0.001. (C) Percentage of Ki-67-positive cells in prostate epithelium of 1-year-old mice. Representative images of Ki-67-stained sections for each genotype are shown. Data are presented as mean ± SEM (n = 6 per genotype). Two equivalently sized regions of interest per lobe (anterior, dorsolateral, and ventral lobes) were quantified, representing at least 10,000 epithelial cells in total per section. Student’s t test was used to determine significant difference between genotypes; *p < 0.05. (D and E) At 40 weeks of age, *Pten*^−/−^ and *Pten*^−/−^;*Ampk*^ACT^ mice were fed a high-fat diet (HFD) for 12 weeks, and prostates were harvested at 1 year of age. (D) Body weight was measured weekly after the start of HFD feeding. Comparable body weights of *Pten*^−/−^ mice on a chow diet are shown at 40 and 52 weeks of age for reference. (E) Quantification of pathological grading from H&E-stained sections of the dorsolateral lobe. Significant difference between the AdCa-free group vs. all other groups is shown using Fisher’s exact test (two-sided p value); ***p < 0.005 (n = 10 for *Pten*^−/−^, n = 7 for *Pten*^−/−^;*Ampk*^ACT^).

In order to assess disease progression, histopathologic analysis was performed on H&E-stained sections from prostates isolated from *Pten*^−/−^ or *Pten*^−/−^;*Ampk*^ACT^ mice at 1 year of age (Figure 1A). By 1 year, approximately 86% of *Pten*^−/−^ mice had developed adenocarcinoma, and a further 7% showed signs of advanced adenocarcinoma. In marked contrast, 60% of *Pten*^−/−^;*Ampk*^ACT^ mice were adenocarcinoma free compared with only 7% in *Pten*^−/−^ mice. Moreover, no *Pten*^−/−^;*Ampk*^ACT^ mice had progressed to the advanced adenocarcinoma stage. There was also a significant reduction in prostate weight in *Pten*^−/−^;*Ampk*^ACT^ mice (Figure 1B). Consistent with these findings, there was a significant reduction in nuclear Ki-67 staining in prostate epithelial cells from *Pten*^−/−^;*AMPK*^ACT^ mice compared with *Pten*^−/−^ mice (Figure 1C). A number of recent studies have linked consumption of HFDs to increased cancer progression, including PCa.^4,15–17^ We wanted to investigate whether AMPK activation could provide protection against HFD-induced PCa progression. We fed *Pten*^−/−^ and *Pten*^−/−^;*Ampk*^ACT^ mice an HFD from 10 months of age for 12 weeks. HFD feeding resulted in a significant increase in body weight in *Pten*^−/−^ and *Pten*^−/−^;*Ampk*^ACT^ mice compared with mice fed a standard chow diet (Figure 1D). Consistent with a recent study,^4^ we found that HFD feeding increased the incidence of advanced PCa in *Pten*^−/−^ mice compared with mice fed a chow diet (4/10 vs. 1/15 mice, respectively; p = 0.041 using chi-square test; Figure 1E compared with Figure 1A). Importantly, AMPK activation provided dramatic protection against disease progression in HFD-fed mice, with none of the *Pten*^−/−^;*Ampk*^ACT^ mice progressing to adenocarcinoma, whereas 90% of the *Pten*^−/−^ mice had progressed to adenocarcinoma or advanced adenocarcinoma (Figures 1E and S1E). Taken together, these findings show that AMPK activation provides a significant protective effect against PCa progression.

### Transcriptional changes following AMPK activation in the *Pten*^−/−^ prostate

**Figure 2.**
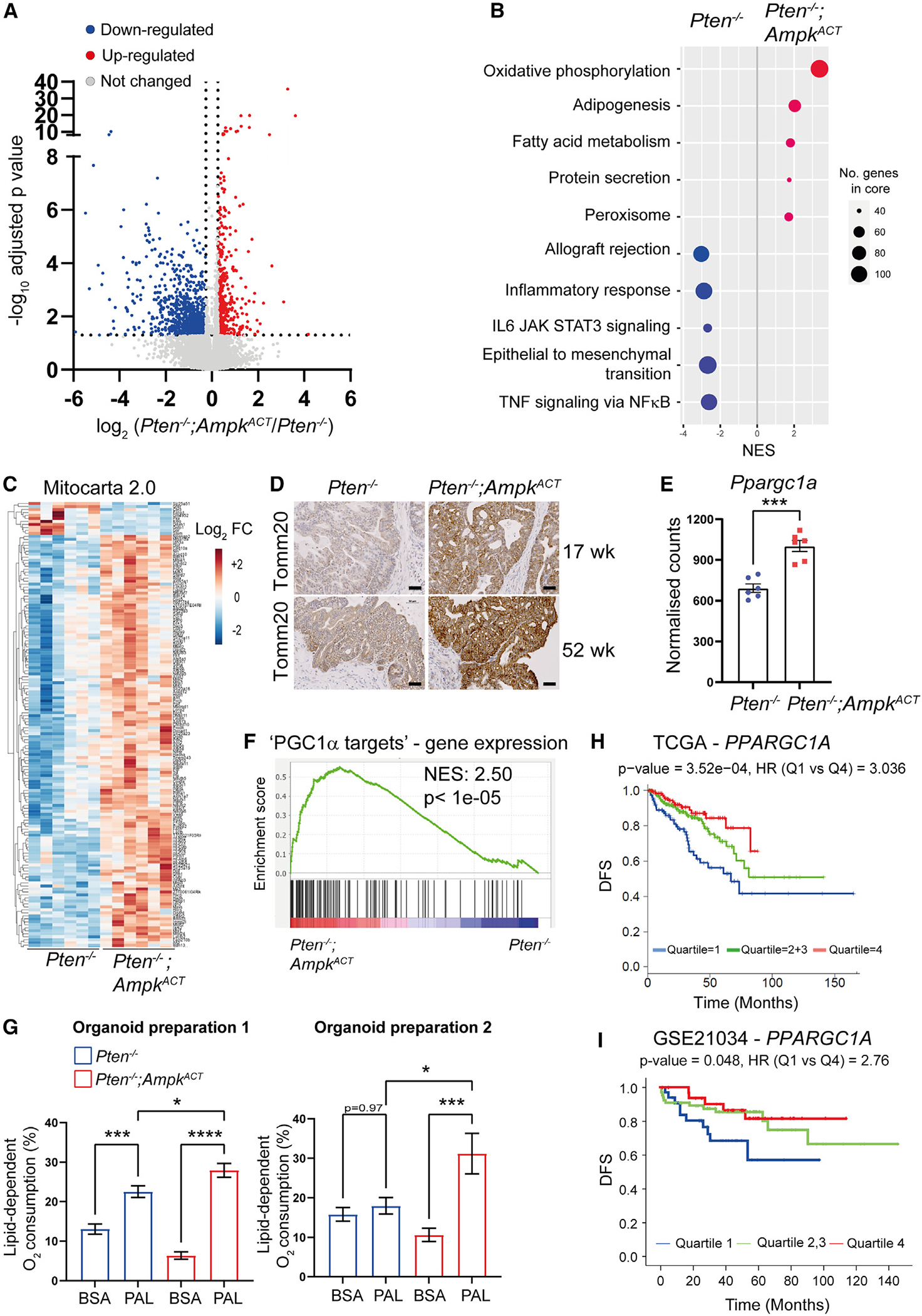
AMPK activation leads to upregulation of Pgc1α and induction of Pgc1α target gene expression RNA sequencing (RNA-seq) was performed on whole prostate tissue from *Pten*^−/−^ and *Pten*^−/−^;*Ampk*^ACT^ mice aged 17 weeks (n = 6 per genotype). (A) Volcano plot summarizing effect of AMPK activation in *Pten*^−/−^ prostates (log_2_ fold change > 1.2, adjusted p < 0.05). (B) Gene set enrichment analysis (GSEA) was performed, and top significant hits are shown (false discovery rate [FDR] q < 0.005 for all hallmark gene sets shown). (C) Heatmap of differentially expressed mitochondrial genes using MitoCarta 2.0. (D) Representative images of TOMM20 staining in dorsolateral prostate sections taken from *Pten*^−/−^ and *Pten*^−/−^;*Ampk*^ACT^ mice aged to either 17 or 52 weeks. Scale bar, 50 μm. (E) *Pgc1α* mRNA (*Ppargc1a*) expression in prostate tissue from *Pten*^−/−^ and *Pten*^−/−^;*Ampk*^ACT^ mice aged 17 weeks. Data are means ± SEM (n = 6 mice per genotype); Student’s t test was used to determine significant differences between genotypes; ***p < 0.005. (F) GSEA plot for the PGC1α target custom gene set (123 genes; Table S1). (G) Lipid-dependent oxygen consumption in prostate organoids generated from *Pten*^−/−^ and *Pten*^−/−^;*Ampk*^ACT^ mice aged 1 year in the presence and absence of exogenous palmitate (PAL; 150 μM). The oxygen consumption rate (OCR) was measured on an Agilent Seahorse XFe96 analyzer before and after addition of etomoxir (100 μM). Lipid-dependent O_2_ consumption (%) was calculated per well as (baseline OCR − etomoxir OCR)/baseline OCR) × 100. Data from two independent organoid preparations from different mice are shown. Data are means ± SEM. n = 10–14 organoids (wells) per genotype. One-way ANOVA with Tukey’s post hoc test was used to determine significant differences between genotypes; *p < 0.05, ***p < 0.005, ****p < 0.001. (H and I) *PGC1α* mRNA (*PPARGC1A*) expression in human PCa separated by disease-free survival (DFS) using the TCGA PRAD (n = 497) dataset (H) and the GSE21034 (n = 150) dataset (I).

To gain mechanistic insight into how AMPK activation might confer protection against disease progression, we characterized prostates from mice aged 17 weeks, when disease progression is less advanced. Consistent with our results from 1-year-old mice, histopathological grading of H&E-stained prostate sections showed that AMPK activation significantly reduced disease progression compared with *Pten*^−/−^ mice (Figure S2A). Ki-67 expression was also significantly reduced in prostate epithelial cells from *Pten*^−/−^;*Ampk*^ACT^ mice (Figure S2B), similar to that seen in 1-year-old mice. We next performed RNA sequencing on RNA isolated from prostate tissue of *Pten*^−/−^ or *Pten*^−/−^;*Ampk*^ACT^ mice aged 17 weeks (Figure 2A). 453 genes were significantly upregulated and 961 genes significantly downregulated in *Pten*^−/−^;*Ampk*^ACT^ mice versus *Pten*^−/−^ mice (log_2_ fold change > 1.2, adjusted p < 0.05). Gene set enrichment analysis revealed that the most significantly enriched hallmark gene set in *Pten*^−/−^;*Ampk*^ACT^ mice was oxidative phosphorylation, whereas several pathways, including inflammatory response, epithelial-to-mesenchymal transition (EMT), and tumor necrosis factor (TNF) signaling via nuclear factor κB (NF-κB), were depleted (Figure 2B). We found a significant downregulation of certain inflammatory mediators, including *Tnf*, *Il1b*, *Ccl2*, and *Cxcl5* (Figure S3A) upon AMPK activation in the *Pten*^−/−^ prostate. To investigate this further, immunohistochemical staining was performed on prostate sections from WT, *Pten*^−/−^, or *Pten*^−/−^;*Ampk*^ACT^ mice, and consistent with decreased expression of certain inflammatory mediators, we found a significant decrease in NF-κB p65 localization in the nucleus (Figure S3B). These data suggest that AMPK activation dampens pro-inflammatory signaling, which has been shown to drive PCa development, progression, and metastasis.^18,19^ No change in apoptosis, as detected by staining for cleaved caspase-3, was seen between tissue sections isolated from *Pten*^−/−^ or *Pten*^−/−^;*Ampk*^ACT^ mice (Figure S3C).

### AMPK activation leads to increased gene expression of PPARGC1A and PGC1α targets

As previously stated, expression of genes relating to oxidative phosphorylation were found to be significantly increased in *Pten*^−/−^;*Ampk*^ACT^ compared with *Pten*^−/−^ prostate (Figure 2B). Further analysis of the transcriptomic data using mouse MitoCarta 2.0^20^ revealed a striking increase in expression of genes encoding mitochondrially localized proteins in prostate tissue from *Pten*^−/−^;*Ampk*^ACT^ mice (Figure 2C). Consistent with this finding, there was a clear increase in expression of TOMM20, a mitochondrial outer membrane protein, in prostate sections taken from 17-week-old or 1-year-old, *Pten*^−/−^;*Ampk*^ACT^ mice (Figure 2D). PGC1α is a transcriptional co-activator that plays an important role in regulating mitochondrial biogenesis and function.^21^ Expression of *Ppargc1a*, the gene encoding PGC1α, was significantly increased in the prostate of *Pten*^−/−^;*Ampk*^ACT^ versus *Pten*^−/−^ mice (Figure 2E). A “PGC1α target” custom gene set was generated from the top 123 genes upregulated upon PGC1α expression in the human PC3 PCa cell line (Table S1) and used as readout of PGC1α activity.^22^ In agreement with the upregulation of *Ppargc1a*, there was significant enrichment of PGC1α target genes in *Pten*^−/−^;*Ampk*^ACT^ versus *Pten*^−/−^ mice (Figure 2F). Because PGC1α has been shown to increase mitochondrial fatty acid oxidation,^21,23,24^ we determined lipid-dependent oxygen consumption in organoids derived from *Pten*^−/−^ and *Pten*^−/−^;*Ampk*^ACT^ mice in the presence or absence of exogenous palmitate, a major substrate for fatty acid oxidation. Lipid-dependent oxygen consumption was calculated by measuring the oxygen consumption rate (OCR) before and after addition of etomoxir. Etomoxir inhibits CPT1, the rate-limiting step in mitochondrial fatty acid uptake, and so the relative contribution of fatty acid oxidation can be calculated by the difference in OCR before and after addition of etomoxir. As shown in Figure 2G, in the presence of exogenous palmitate, fatty acid oxidation was significantly higher in 2 independent organoid cultures derived from *Pten*^−/−^;*Ampk*^ACT^ mice compared with *Pten*^−/−^ mice. Interestingly, the difference between lipid-derived oxygen consumption in the presence of palmitate versus bovine serum albumin (BSA) alone was much greater in *Pte*n^−/−^;*Ampk*^ACT^ organoids compared with *Pten*^−/−^ organoids (mean difference 21.57 ± 6.00 [*Pten*^−/−^;*Ampk*^ACT^] vs. 9.47 ± 5.37 [*Pten*^−/−^] for organoid prep 1; 20.60 ± 12.43 [*Pten*^−/−^;*Ampk*^ACT^] vs. no difference [*Pten*^−/−^] for organoid prep 2). This was in part due to the higher level of lipid-derived oxygen consumption seen in the absence of palmitate in organoids from *Pten*^−/−^ mice (Figure 2G). One possibility that could explain this observation is that *Pten*^−/−^ organoids have higher levels of stored lipids available for fatty acid oxidation compared with *Pten*^−/−^;*Ampk*^ACT^ organoids. A previous study provided evidence supporting an important role of PGC1α acting as a metabolic co-regulator suppressing PCa progression.^22^ Torrano et al.^22^ defined a tumor-suppressive catabolic state induced upon PGC1α expression, characterized by increased fatty acid oxidation and decreased lipogenesis. Consistent with this finding, *PPARGC1A* expression was found to be progressively lost during PCa progression and positively correlate with disease-free survival (DFS). An updated version of the correlation between *PPARGC1A* expression and DFS, based on primary tumors using The Cancer Genome Atlas (TCGA) prostate adenocarcinoma (PRAD) dataset (n = 497),^25^ is shown in Figure 2H. In addition, analysis of *PPARGC1A* expression using a dataset that includes metastatic disease^26^ also showed a positive correlation with DFS (Figure 2I).

### AMPK activation decreases steady-state levels of neutral storage lipids

**Figure 3.**
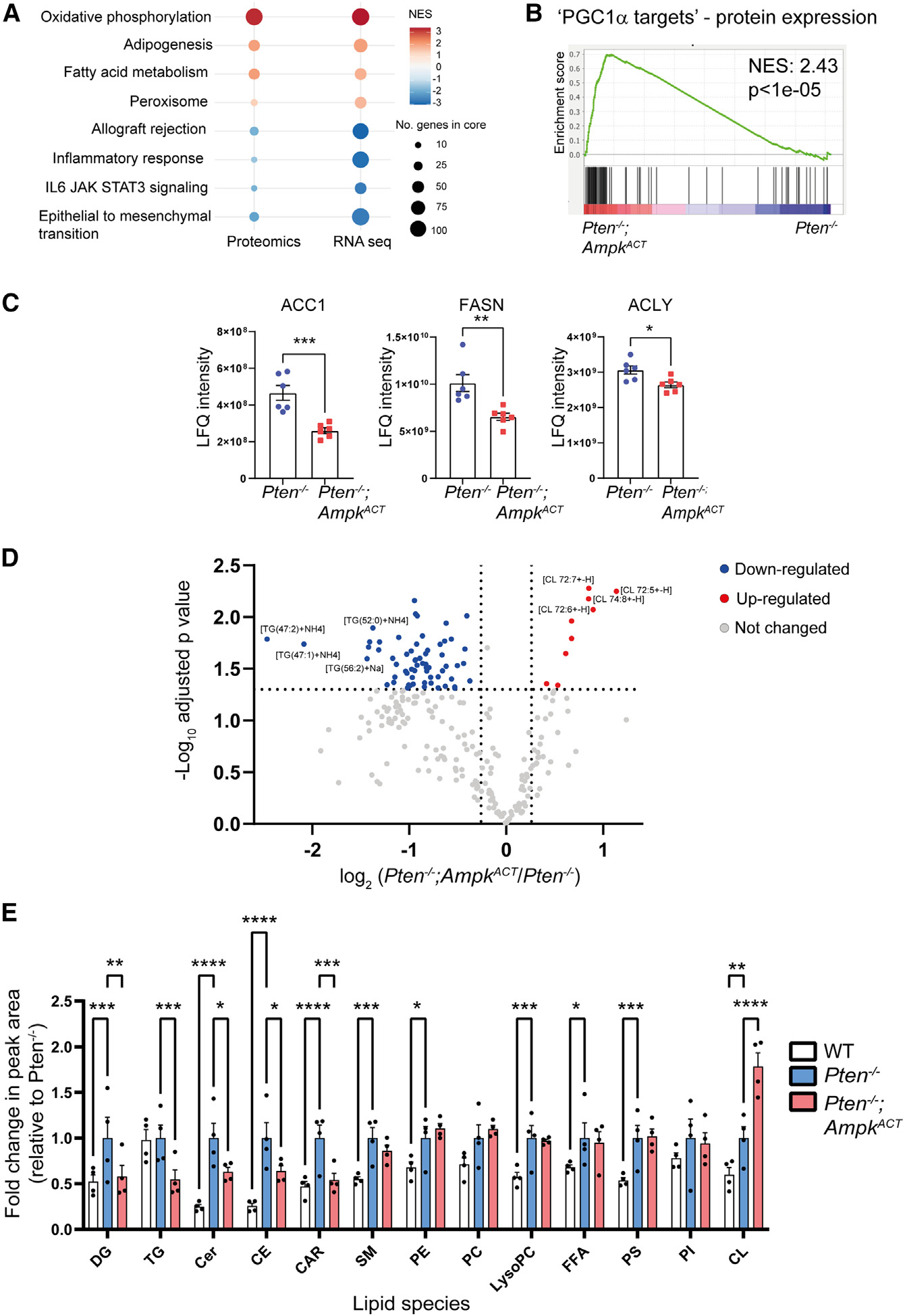
AMPK activation significantly impacts lipid homeostasis in the *Pten*^−/−^ prostate (A) Proteomics analysis was performed on prostate tissue from *Pten*^−/−^ and *Pten*^−/−^;*Ampk*^ACT^ mice aged 17 weeks (n = 6 per genotype). Comparison between RNA-seq GSEA (FDR q < 0.005) and proteomics (FDR q < 0.1) protein set enrichment analysis is shown. (B) Protein set enrichment analysis plot for PGC1α target proteins derived from the custom gene set (123 genes; Table S1) based on protein expression data for 81 proteins identified for *Pten*^−/−^;*Ampk*^ACT^ versus *Pten*^−/−^. (C) Protein expression of key enzymes involved in fatty acid synthesis. Data are means ± SEM. Student’s t test was used to determine significant differences between genotypes; *p < 0.05, **p < 0.01, ***p < 0.005. (D) Lipidomics analysis was performed on prostate tissue from WT, *Pten*^−/−^, and *Pten*^−/−^;*Ampk*^ACT^ mice aged 17 weeks (n = 4 per genotype). A volcano plot summarizing the effect of AMPK activation in the *Pten*^−/−^ prostate on individual lipid species (fold change > 1.2; adjusted p < 0.05) is shown. (E) Abundance of different lipid classes in WT, *Pten*^−/−^, and *Pten*^−/−^;*Ampk*^ACT^ prostates. Data are plotted as fold change relative to *Pten*^−/−^ and are the means ± SEM (n = 4 per genotype). Two-way ANOVA with uncorrected Fisher’s LSD (least significant difference) was used to determine significant differences between groups; *p < 0.05, **p < 0.01, ***p < 0.005, ****p < 0.001. NES, normalized enrichment score; ACC1, acetyl-CoA carboxylase 1; ACLY, ATP-citrate lyase; FASN, fatty acid synthase; DG, diglyceride; TG, triglyceride; Cer, ceramide; CE, cholesteroyl esters; CAR, acylcarnitine; SM, sphingomyelin; PE, phosphatidylethanolamine; PC, phosphatidylcholine; FFA, free fatty acid; PS, phosphatidylserine; PI, phosphatidylinositol; CL, cardiolipin.

In parallel with the transcriptomic analysis, we carried out a proteomics screen to determine changes in protein abundance in prostates isolated from *Pten*^−/−^and *Pten*^−/−^;*Ampk*^ACT^ mice (Table S2). Although protein coverage was less comprehensive compared with the transcriptomics analysis, there was a high degree of overlap between protein and mRNA expression (Figure 3A). In agreement with gene expression data, a significant enrichment of PGC1α target protein expression was determined in *Pten*^−/−^;*Ampk*^ACT^ versus *Pten*^−/−^ mice (Figure 3B). Further analysis of the proteomics data from 17-week-old mice revealed significant reductions in the abundance of key proteins involved in fatty acid biosynthesis (ACC1, fatty acid synthase [FASN], and ATP-citrate lyase [ACLY]) from Pten^−/−^ prostates with AMPK activation (Figure 3C). Of note, there was no significant difference in their gene expression, suggesting that the changes in protein level occur post-transcriptionally. These results, along with increased ACC phosphorylation (Figure 1A), are consistent with decreased lipogenic potential in *Pten*^−/−^;*Ampk*^ACT^ versus *Pten*^−/−^ prostates. To determine whether there was any impact on lipid homeostasis as a result of the reduction in lipogenic protein expression and/or the increase in fatty acid oxidation, we performed a global lipidomics screen using mass spectrometry to investigate lipid content in mouse prostate tissue. AMPK activation resulted in significant changes in lipids compared with prostate tissue from *Pten*^−/−^ mice, primarily decreasing the abundance of several lipid species (Table S3; 64 significantly downregulated ions versus 9 upregulated from 257 uniquely identified ions; Figure 3D). We compared changes in the total abundance of different lipid classes from WT, *Pten*^−/−^, and *Pten*^−/−^;*Ampk*^ACT^ mice (Figure 3E). The abundance of most lipid classes was significantly increased in *Pten*^−/−^ mice compared with WT mice. Notably, AMPK activation significantly decreased the levels of triglycerides, diglycerides, ceramides, cholesteryl esters, and acyl-carnitine in *Pten*^−/−^ mice. These results support a model where inhibition of *de novo* lipogenesis, together with increased fatty acid oxidation, as a result of AMPK activation leads directly to decreased lipid levels and, in particular, triglycerides and diglycerides, the major storage lipids. In contrast, cardiolipin was significantly increased in prostate tissue from *Pten*^−/−^;*Ampk*^ACT^ compared with *Pten*^−/−^ mice. Given that cardiolipin is almost exclusively found in the inner membrane of mitochondria,^27^ this finding is consistent with the increase in mitochondria in prostates from *Pten*^−/−^;*Ampk*^ACT^ mice.

### Pharmacological activation of AMPK induces a catabolic program in human PCa cells

**Figure 4.**
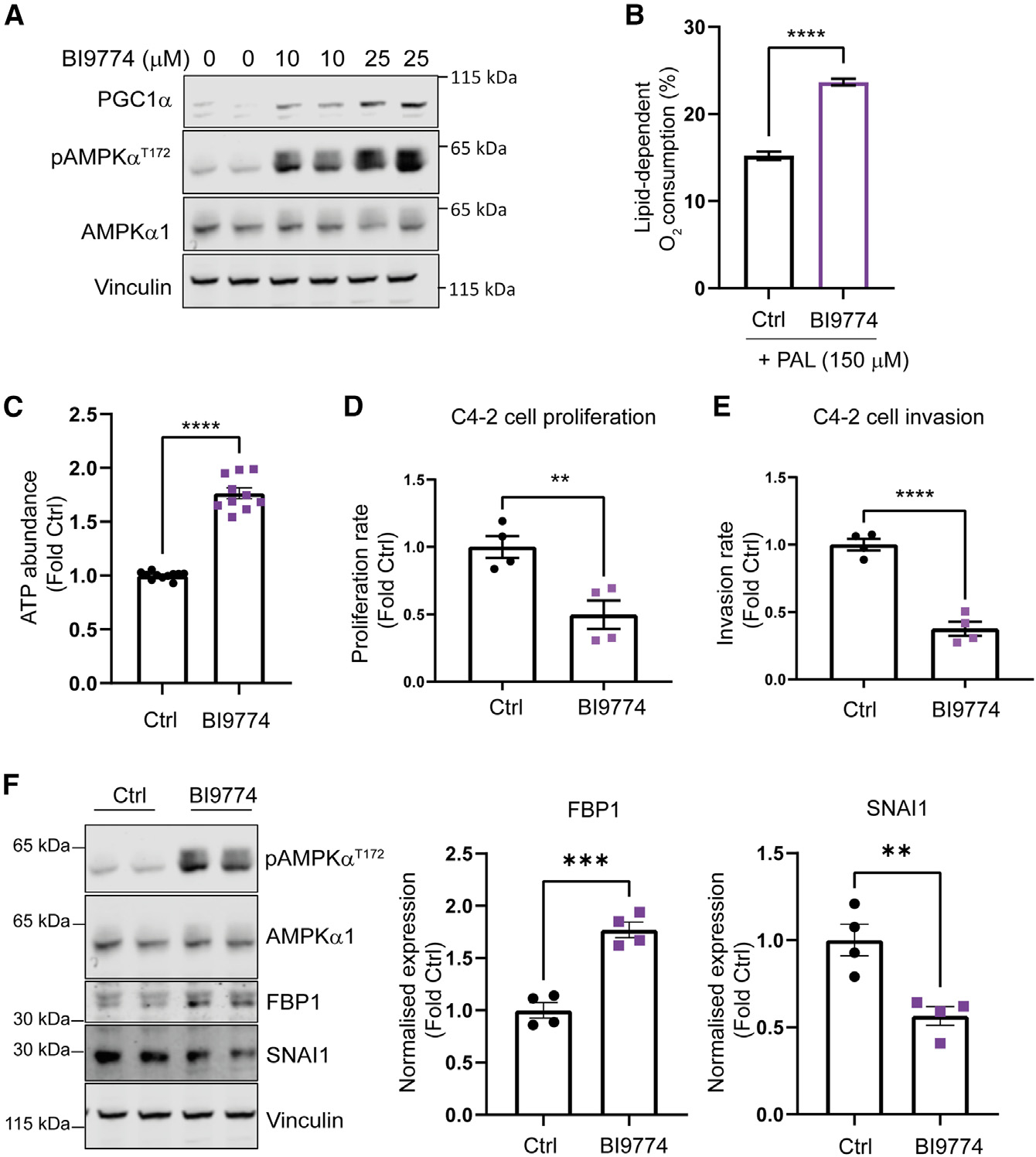
AMPK activation leads to PGC1α induction in a human CRPC cell model and inhibits PCa cell proliferation and invasion (A) Castration-resistant prostate cancer (CRPC) C4–2 cells were treated with the AMPK activator (BI9774; 0, 10, and 25 μM) for 1 week, and PGC1α expression was determined by western blotting. (B) Lipid-dependent oxygen consumption for C4–2 cells treated for 1 week with or without 10 μM BI9774 was determined in the presence of exogenous PAL (150 μM). The OCR was measured on an Agilent Seahorse XFe96 analyzer before and after addition of etomoxir (50 μM). Lipid-dependent O_2_ consumption (%) was calculated per well as (baseline OCR − etomoxir OCR)/baseline OCR) × 100 (n = 11 per group). (C–E) ATP abundance was determined in C4–2 cells cultured with or without BI9774 (10 μM) for 7 days (n = 10 per group). Proliferation rate (D) and invasion rate (E) were determined for C4–2 cells treated as in (C) (n = 4 per group). (F) Western blot analysis of FBP1 and SNAI1 expression in C4–2 cells treated as in (C) and quantification (n = 4 per group). In all cases, data shown are means ± SEM, and statistical significance was determined using Student’s t test. **p < 0.01, ***p < 0.005, ****p < 0.001.

Over the last few years a number of potent and selective direct small-molecule activators of AMPK have been developed, opening up the therapeutic potential of AMPK activation.^6^ We found PGC1α protein expression to be significantly upregulated in a dose-dependent manner in a human CRPC cell line (C4–2 cells) following chronic treatment with the direct AMPK activator BI9774^10^ (Figure 4A). Upregulation of PGC1α expression following AMPK activation is consistent with the increase in PGC1α expression we observed in *Pten*^−/−^;*Ampk*^ACT^ mice (Figure 2E). The expression of mitochondrial proteins was also found to be increased in cells treated with BI9774 (Figure S4A). We measured the contribution of fatty acid oxidation to overall oxygen consumption in C4–2 cells maintained in the presence or absence of BI9774 for 7 days and in the presence of exogenous palmitate. Similar to our finding using organoid cultures, activation of AMPK with BI9774 caused a significant increase in fatty acid oxidation (Figure 4B). Again, similar to our findings in prostate organoids, AMPK activation led to a significant increase in levels of ACC phosphorylation, thereby inhibiting fatty acid synthesis and concomitantly increasing fatty acid oxidation (Figure S4B). Consistent with an AMPK-induced catabolic cellular state, we found a significant increase in intracellular ATP levels of around 1.7-fold in C4–2 cells following 7-day treatment with BI9774 (Figure 4C). Blocking fatty acid oxidation with etomoxir reduced, but did not abolish, the BI9774-induced increase in ATP (Figure S4C). This finding is consistent with the hypothesis that AMPK activation decreases ATP utilization and increases ATP production. Importantly, we found that these metabolic changes correlated with reduced CRPC cell proliferation and invasion (Figures 4D and 4E).

EMT allows cancer cells to increase their motility and invasiveness. Part of this process includes loss of tumor suppressor fructose-1,6-biphosphatase (*FBP1*) gene expression and induction of the transcription factor SNAI1.^28,29^ *FBP1* loss mediated by SNAI1 leads to changes in metabolism, resulting in increased glycolysis.^28^ In C4–2 cells, AMPK activation by BI9774 resulted in a significant decrease in SNAI1 protein expression and a concomitant increase in FBP1 protein levels (Figure 4F). Consistent with the cell data, we also saw increased mRNA and protein expression of *Fbp1* in prostate tissue from *Pten*^−/−^;*Ampk*^ACT^ mice compared with *Pten*^−/−^ mice (Figure S4D). Although we were unable to detect SNAI1 protein in our proteomics analysis of prostate tissue, mRNA expression was significantly decreased in *Pten*^−/−^;*Ampk*^ACT^ mice compared with *Pten*^−/−^ mice (Figure S4E). These findings suggest that the reduction in cell invasiveness following AMPK activation could be in part due to reduced SNAI1 expression and increased FBP1 expression.

### Identification of an AMPK-regulated-cell cycle gene network

**Figure 5.**
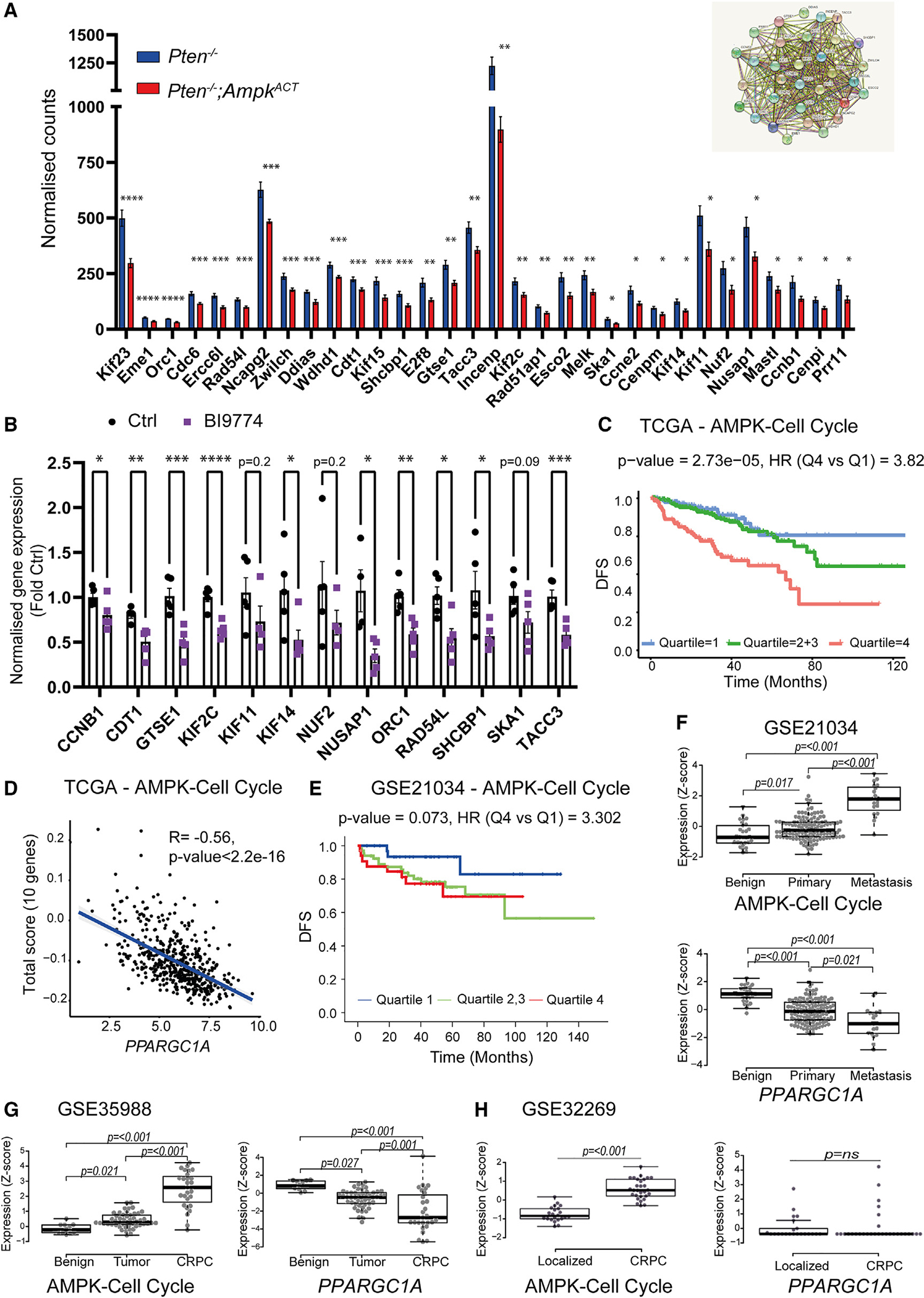
AMPK activation leads to downregulation of a transcriptional network required for PCa progression (A) Expression of 32 genes negatively correlated with DFS in prostate tissue from *Pten*^−/−^ and *Pten*^−/−^;*Ampk*^ACT^ mice aged 17 weeks (mean ± SEM, n = 6 per genotype). Statistical significance was determined using Student’s t test. *p < 0.05, **p < 0.01, ***p < 0.005, ****p < 0.001. The inset shows the interacting network of the 32 genes using STRING. (B) Quantification of mRNA expression using qRT-PCR for a subset of 13 “cell cycle” genes in human CRPC C4–2 cells treated with or without BI9774 (10 μM) for 7 days. Expression is normalized to *UBC* expression and shown as fold change relative to untreated cells. Data shown are means ± SEM (n = 4, 5), and statistical significance was determined using Student’s t test. *p < 0.05, **p < 0.01, ***p < 0.005, ****p < 0.001. (C) Gene expression of an “AMPK-cell cycle” curated gene set (10 genes) in human PCa separated by disease free survival (DFS) using the TCGA PRAD (n = 497) dataset. The inset shows tabulated data for hazard ratio (HR) between Q4 and Q1 and p value. (D) Correlation of gene expression with *PGC1α* (*PPARGC1A*) gene expression using the TCGA PRAD (n = 497). The inset shows tabulated data for rank correlation coefficient (R) value and p value. (E–H) AMPK-cell cycle gene expression in human PCa separated by DFS using the GSE21034 (n = 150) dataset. (F–H) *Z* scores comparing mRNA expression of AMPK-cell cycle gene signature and *PPARGC1A* between benign tissue, localized cancer, and either metastatic or CRPC from the (F) GSE21034 (benign, n = 29; localized cancer, n = 131; metastasis, n = 19), (G) GSE35988 (benign, n = 12; tumor, n = 49; CRPC, n = 27), and (H) GSE32269 (localized, n = 22; CRPC, n = 29) datasets. Data are presented as *Z* score of log2 expression values. Significance was tested using Kruskal-Wallis non-parametric test.

Activation of AMPK has been shown to cause cell-cycle arrest in G1^7,30^ or G2,^31,32^ inhibiting cell proliferation, although the mechanism by which AMPK triggers cell-cycle arrest is not known. We used the STRING database^33^ to search for interacting networks present in the genes that were significantly downregulated in prostates from 17-week-old *Pten*^−/−^;*Ampk*^ACT^ mice compared with *Pten*^−/−^ mice. One of the top networks to emerge using the Markov cluster algorithm^34^ included 38 genes that were associated with regulation of the cell cycle. This cluster includes proteins involved at different stages of the cell cycle, including G1/S phase transition (e.g., cyclin E2) and G2/M transition (e.g., cyclin B1). Using the TCGA PRAD (n = 497, primary PCa gene expression) dataset and the CANCERTOOL interface,^35^ expression of the 38 “cell cycle” genes was correlated with DFS (Table S4). 32 of the 38 “cell cycle” genes were significantly negatively correlated with DFS, and the expression of these 32 genes in prostate tissue from *Pten*^−/−^ versus *Pten*^−/−^;*Ampk*^ACT^ mice is shown in Figure 5A. Interestingly, expression of 31 of these 32 genes was significantly negatively correlated with *PPARGC1A* expression (Table S5). To determine whether AMPK activation had an effect on expression of these genes in human PCa cells, we measured the expression of the top 14 cell cycle genes that were most strongly correlated with DFS (Table S4) in C4–2 cells treated with or without BI9774 for 1 week (Figure 5B). Expression of 10 genes was significantly decreased following BI9774 treatment (Figure 5B). We were unable to detect expression of EME1 in C4–2 cells in either condition. We used the 10 significantly changed genes (CCNB1, CDT1, GTSE1, KIF2C, KIF14, NUSAP1, ORC1, RAD54L, SHCBP1, and TACC3) to curate an “AMPK-cell cycle” gene signature. The association of these 10 genes with DFS using the TCGA PRAD dataset is shown in Figure 5C and the correlation with *PPARGC1A* gene expression in Figure 5D. In parallel, the same analyses were performed using the 38 genes present in the cell cycle network; the results are shown in Figures S5A and S5B. Furthermore, the association of DFS with the AMPK-cell cycle genes in a dataset including metastatic disease^26^ is shown in Figure 5E. Expression of the AMPK-cell cycle genes in the different stages of PCa (benign tissue, localized cancer, and advanced disease) is shown in Figures 5F–5H) alongside expression of *PPARGC1A*. These analyses consistently show that the AMPK-cell cycle genes are positively correlated with disease progression, whereas *PPARGC1A* expression is inversely correlated with PCa.

### PGC1α is an important mediator of the effects of AMPK activation in PCa cells

To further explore the role of PGC1α in mediating the effects of AMPK activation in PCa cells, we transiently overexpressed PGC1α in C4–2 cells (Figure S5C). Similar to AMPK activation, overexpression of PGC1α led to an increase in ATP levels, decreased cell proliferation, and decreased cell invasion (Figure S5). Transient PGC1α overexpression also significantly decreased 4 of the 10 “AMPK-cell cycle” genes (KIF14, NUSAP1, RAD54L, and TACC3; Figure S5G). Our findings suggest that increased PGC1α expression is sufficient to mediate some of the effects of AMPK activation in PCa cells and reveal an intriguing link between AMPK activation, PGC1α, and suppression of genes involved in regulation of the cell cycle.

### Pharmacological activation of AMPK reduces tumor growth in a CRPC xenograft model

**Figure 6.**
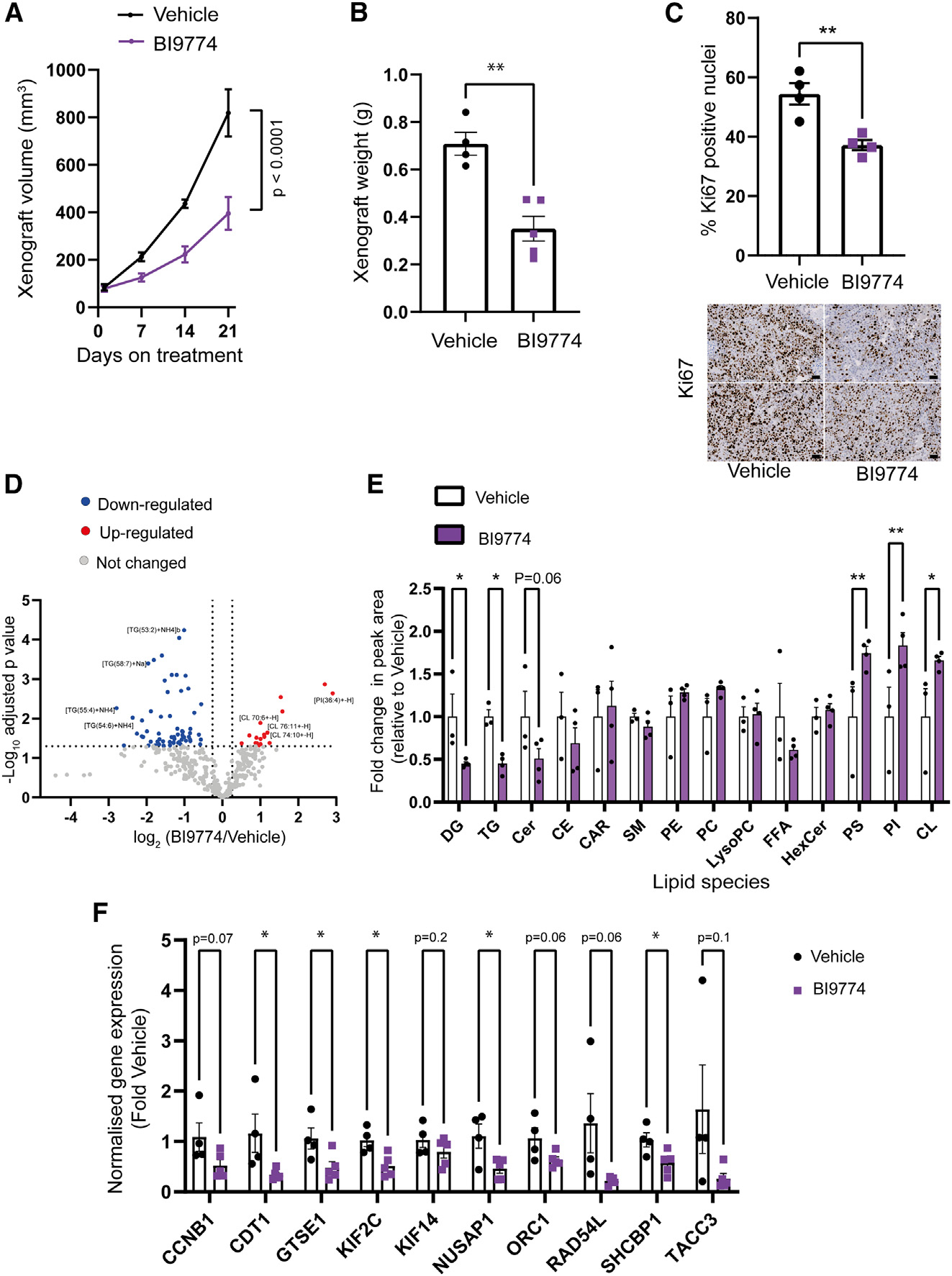
Pharmacological activation of AMPK inhibits tumor growth in a xenograft model of CRPC (A) Average tumor volume during 21-day treatment with the AMPK activator BI9774 (30 mg/kg, daily oral gavage) or vehicle control. Significant differences in growth rate were calculated using linear regression analysis. (B) Xenograft weight was determined upon harvest. Data are means ± SEM (n = 4–5 mice per group). (C) Percentage of Ki-67-positive cells in xenograft tumors from mice treated with or without BI9774 (n = 4 per condition, at least 10,000 cells counted per section). Below, representative images of Ki-67-stained sections taken from xenografts isolated from mice treated with either vehicle or Bi9774 are shown (scale bar, 50 μm). Lipidomics analysis was performed on xenograft tissue from mice treated with either vehicle (n = 3) or BI9774 (n = 4). (D) Volcano plot summarizing the effect of pharmacological AMPK activation in the C4–2b xenograft model on individual lipid species (fold change > 1.2, adjusted p < 0.05). (E) Abundance of different lipid classes in xenografts isolated from vehicle- or BI9774-treated mice. Data are plotted as fold change relative to vehicle and are the means ± SEM (n = 3 (vehicle treated) and 4 (BI9774 treated)). Two-way ANOVA with uncorrected Fisher’s LSD was used to determine significant differences between groups; *p < 0.05, **p < 0.01. (F) Quantification of mRNA expression for a subset of 10 “AMPK-cell cycle” genes in xenografts isolated from vehicle- or BI9774-treated mice. Expression is normalized to *UBC* expression and shown as fold change relative to xenografts from mice treated with vehicle. Data shown are means ± SEM (n = 3–5 xenografts), and statistical significance was determined using Student’s t test. *p < 0.05, **p < 0.01, ***p < 0.005, ****p < 0.001. HexCer, hexosylceramide.

To investigate whether pharmacological AMPK activation could act as a therapeutic intervention in PCa, we made use of the C4–2b xenograft model of CRPC. Immune-compromised non-obese diabetic (NOD) severe combined immunodeficiency (SCID) gamma (NSG) mice were inoculated with C4–2b cells, and tumors were allowed to grow to 90 mm^3^ before being randomized into BI9774 or vehicle treatment groups. Mice were dosed daily by oral gavage for 3 weeks. Consistent with our findings using genetic and *in vitro* models of AMPK activation in PCa, BI9774 treatment significantly reduced tumor growth compared with vehicle controls (Figure 6A), and this corresponded to reduced tumor weight upon harvest (Figure 6B). Cell proliferation, as determined by staining with Ki67, was also significantly reduced in tumors taken from BI9774-treated mice (Figure 6C). Lipidomics analysis revealed significant changes in lipid species between C4–2b tumors taken from vehicle- and BI9774-treated mice (Figure 6D; Table S6). Similar to our findings in mouse prostate tissue, BI9774 treatment led primarily to a decrease in lipid content (62 significantly downregulated ions versus 17 upregulated from 370 uniquely identified ions; Figure 6D; Table S6). Overall, the changes in abundance of different lipid classes in response to BI9774 treatment were similar to those seen in prostate tissue from *Pten*^−/−^;*Ampk*^ACT^ mice relative to *Pten*^−/−^ mice (compare Figures 6E and 3E), with significant reductions in triglycerides and diglycerides and a significant increase in cardiolipin. Gene expression analysis revealed that expression of 5 of the 10 “AMPK-cell cycle” genes (CDT1, GTSE1, KIF2C, NUSAP1, and SHCBP1) was significantly decreased in xenograft tissue isolated from mice treated with BI9774 (Figure 6F). Overall, pharmacological AMPK activation in the xenograft model of CRPC closely mirrors the tumor-suppressive effect of genetic AMPK activation in the *Pten*^−/−^ mouse model. AMPK activation leads to dramatic changes in metabolism, switching from an anabolic to a catabolic program. This catabolic state inhibits prostate cancer disease progression and is linked with induction of PGC1α, decreased cell proliferation, and decreased cell invasiveness. Importantly, we show a strong negative correlation between AMPK-regulated cell cycle genes and *PPARGC1A* expression in human prostate cancer. Taken together, our findings strongly support the use of AMPK activators for treatment of PCa.

## DISCUSSION

We previously reported that deletion of *Prkab1*, encoding the β1 subunit of AMPK, increased cancer progression in *Pten*^−/−^ mice,^7^ suggesting that AMPK activation could have a tumor-suppressive effect on PCa *in vivo*. In the current study, we now provide direct evidence that this is the case. Genetic activation of AMPK in prostate epithelial cells *in vivo* provided a strong protective effect on PCa progression in *Pten*^−/−^ mice. Additionally, we show that pharmacological AMPK activation using a potent and specific direct AMPK activator, BI9774, reduces tumor growth in a xenograft model of CRPC. AMPK activation resulted in an increase in Pgc1α expression, together with a striking increase in the expression of mitochondrial genes and mitochondrial biogenesis. AMPK is an upstream activator of PGC1α,^36^ and many of the effects of AMPK on mitochondrial function are thought to be mediated through activation of PGC1α. Importantly, PGC1α has been shown previously to suppress PCa progression and metastasis by promoting a catabolic metabolic program characterized by increased fatty acid oxidation, decreased lipogenesis, and increased intracellular ATP.^22^ Consistent with this previous study, we show that overexpression of PGC1α in human PCa cells increases ATP levels, lowers the rate of cell proliferation, and decreases cell invasiveness. These findings imply that increased PGC1α expression is sufficient to mediate the effect of AMPK activation on these processes.

In addition to its effects on PGC1α, AMPK phosphorylates and inactivates ACC, leading to a reduction in malonyl-CoA and increased uptake of fatty acids into mitochondria via CPT1. These combined effects can account for the increase in fatty acid oxidation that we observed in prostate organoids isolated from *Pten*^−/−^;*Ampk*^ACT^ mice compared with *Pten*^−/−^ mice. Similar effects were seen in the human CRPC PCa C4–2 cell line following pharmacological activation of AMPK, and AMPK activation was found to lead to an increase in intracellular ATP levels. Importantly, this switch to a catabolic metabolic state with increased ATP production is not associated with increased cellular proliferation or invasiveness. The finding that a switch from an anabolic to a catabolic metabolic program provides protection against PCa tumor growth is supported by our observation that there is a switch from ACC2 to ACC1 gene expression in normal to cancerous prostate tissue in humans (Figure S6). High ACC1 expression is found in tissues with high rates of *de novo* lipogenesis, such as liver and adipose tissue, whereas ACC2 expression is found primarily in oxidative tissues, such as heart and skeletal muscle.^37^ We note that ACC1 is an androgen-responsive gene, and therefore increased ACC1 expression in androgen receptor-dependent prostate cancers would be anticipated. However, the mechanism for decreased expression of ACC2 in these cancers is not clear. The switch from ACC2 to ACC1 expression in human PCa supports the hypothesis of a switch from an oxidative to a lipogenic program during tumorigenesis, required for driving PCa progression.

High rates of *de novo* lipogenesis are a common feature of highly proliferating cancer cells and important for PCa cell growth.^4,38–40^ In addition, other studies have shown that consuming diets high in fat promotes aggressive disease progression in PCa,^4,16^ indicating that exogenous lipids contribute to cancer cell growth in addition to lipids generated through *de novo* lipogenesis. AMPK inhibits *de novo* lipogenesis through phosphorylation and inactivation of ACC, thereby slowing PCa cell growth. Furthermore, the protection against disease progression by AMPK activation was also maintained in 1-year-old mice fed an HFD for the final 12 weeks. In the mouse model we used, AMPK activation is restricted to prostate epithelial cells, so the protective effect we observe originates from changes in these cells. It is interesting to speculate whether more widespread activation of AMPK could have further protective effects on cancer progression, mediated by changes in whole-body metabolism in response to feeding an HFD.^11,41^ Lipidomics analysis of mouse prostate tissue revealed a significant reduction in the abundance of a number of lipid classes, including the major neutral storage lipids (triglycerides and diglycerides), in *Pten*^−/−^;*Ampk*^ACT^ mice compared with *Pten*^−/−^ mice. Similarly, pharmacological AMPK activation led to a reduction in triglyceride and diglyceride content in PCa xenograft tumors. A recent study reported that neutral lipid accumulation in PCa tumors, either through *de novo* lipogenesis or increased dietary lipid intake, drives advanced PCa progression.^4^ Moreover, the degree of neutral lipid accumulation in the tumors positively correlated with disease aggressiveness. In the same study, the authors showed that addition of palmitate or oleate to PCa cells in culture increased cell invasiveness, and this correlated with an increase in intracellular triglyceride accumulation. Our results are consistent with these findings, showing that decreased triglyceride and diglyceride accumulation in response to AMPK activation is associated with dramatically improved disease outcome. This reduction in neutral lipid accumulation is likely to result from the combination of decreased *de novo* lipogenesis together with increased fatty acid oxidation. Importantly, we show that an increase in fatty acid oxidation mediated through increased AMPK-PGC1α signaling does not fuel anabolic processes such as increased cell proliferation or invasiveness. At present, the downstream consequences of increased ATP production in PCa cells following AMPK activation remain unknown and beyond the scope of the current study.

We found that chronic AMPK activation led to reduced cell proliferation *in vivo* and in PCa cells in culture. One mechanism by which AMPK restricts cell growth is by slowing the cell cycle, mediated at least in part by preventing cells from entering S phase^7,30^ or mitosis^31,32^ of the cell cycle. We identified a network of 32 genes that are downregulated in mouse prostate in response to chronic AMPK activation that are involved in cell cycle regulation and negatively correlate with DFS in human PCa. These genes encode proteins that function at different stages of the cell cycle, raising the possibility that chronic AMPK activation can regulate multiple stages of the cell cycle in parallel, not just the G1-to-S transition or G2-to-M transition, as reported previously.^7,30–32^ The expression of a subset of these genes (10 of 32) was decreased by pharmacological activation of AMPK in C4–2 cells, and we termed this subset of genes the “AMPK-cell cycle” gene network because their expression is significantly changed following AMPK activation in mouse and human PCa cells. A previous study reported that overexpression of PGC1α in PC3 cells increased the proportion of cells in G1 with a concomitant decrease in the proportion of cells in S phase.^22^ Intriguingly, we found that there was a negative correlation between PGC1α gene expression and this cell-cycle-regulatory gene network in human primary PCa tumors. Furthermore, we found that overexpression of PGC1α in C4–2 cells was sufficient to decrease expression of some of the AMPK-cell cycle genes. Taken together, our results suggest a direct link between AMPK, PGC1α, and the cell cycle. Further work is needed to investigate whether this link occurs in other PCa cell lines or in other types of cancer cells. Nonetheless, our findings raise the possibility that PGC1α gene expression, together with the AMPK-cell cycle genes, could be used as a surrogate marker for AMPK activation, at least in PCa. This would provide a valuable readout for future studies because determining the level of AMPK expression and activity in cancer tissues is hampered by the existence of multiple subunit isoforms together with extensive post-translational regulation of its activity.^6^

The genetic gain-of-function AMPK mutant used in our study provided us with an *in vivo* model for exploring the effects of chronic AMPK activation in PCa. Our findings reveal a number of diverse and beneficial effects of AMPK activation. We saw a reduction in cell invasiveness associated with decreased Snai1 expression and increased Fbp1 expression. Previous studies have found a reciprocal relationship between SNAI1 and FBP1 expression,^28,29^ with increased SNAI1 expression associated with EMT and decreased FBP1 expression associated with increased glycolysis to drive anabolic pathways.^28^ In addition, AMPK activation reduced inflammation via reduced NF-κB signaling. Relevant to our study, a number of previous studies have shown that PGC1α can inhibit NF-κB signaling through multiple mechanisms, including decreased phosphorylation of p65.^42,43^

Overall, our findings show that AMPK activation provides a strong protective effect against PCa progression at early and late stages of the disease. Our studies reveal multiple mechanisms for the protective effect of AMPK activation, including increased PGC1α expression and a switch from anabolic to catabolic metabolism. As far as we are aware, and in contrast to AMPK, there are no direct activators targeting PGC1α, excluding it as a therapeutic target. Given the multifaceted nature of the beneficial effects of AMPK activation in PCa, there is a compelling rationale for therapeutic targeting of AMPK in this disease. Related to this, metformin, a drug widely used for treatment of type 2 diabetes and an indirect activator of AMPK,^6^ is currently being evaluated in PCa. The STAMPEDE (Systemic Therapy in Advancing or Metastatic Prostate Cancer: Evaluation of Drug Efficacy) trial is investigating whether addition of metformin has beneficial effects in men with high-risk locally advanced or metastatic PCa.^44^ However, as far as we are aware, it is not known whether metformin leads to activation of AMPK in the prostate, and so other approaches may need to be considered. A number of potent and selective small-molecule direct activators of AMPK have been well characterized and show good bioavailability. Recently, Poxel announced the results of a phase 1b clinical trial for treatment of non-alcoholic fatty liver disease, indicating the potential for safe, therapeutic targeting of AMPK.^45^ AMPK phosphorylates at least 100 targets involved in regulating a number of diverse cellular pathways,^46^ and we lack a complete understanding of all of the effects of activating AMPK *in vivo*. This incomplete knowledge extends to the role of AMPK in cancer, and there are studies suggesting either a tumor-suppressive effect of AMPK activation or a tumor-promoting effect.^6^ An important outcome of our current study is that it provides strong support in favor of a beneficial effect of AMPK activation in PCa. As with any potential treatment, the ultimate test will be to undertake clinical trials, and the finding that a direct AMPK activator is safe and well tolerated in humans opens up this crucial opportunity.

### Limitations of the study

A limitation of this study is that, in the genetic gain-of-function mouse model we used, AMPK activation occurs at the same time as deletion of Pten. It is not possible for us to determine to what extent AMPK activation prevents PCa disease progression versus cancer initiation in this model. One way to address this issue would be to activate AMPK pharmacologically in a mouse model of late-stage PCa, and in this regard, it is interesting to note that a mouse model of PCa metastasis has been described recently.^47^ We do show that pharmacological activation of AMPK significantly slows tumor growth in a xenograft model, suggesting that AMPK can prevent disease progression. Furthermore, we did not assess the impact of AMPK activation in combination with hormone therapies, such as enzalutamide, and this is an issue that could be pursued in subsequent studies. In our “omics” studies, we used total prostate tissue comprising all lobes. We recognize that the “omics” profiles of the different lobes may be distinct and affected by the penetrance of the genetic alteration, and so it is possible that our approach may have missed lobe-specific changes in these profiles. Another important limitation of our current study is that we have not been able to address directly the contribution of PGC1α in the protective effect of AMPK activation. Despite significant effort, we were unable to generate PCa cells lacking PGC1α, precluding us from carrying out this aspect of the work. However, our finding that overexpression of PGC1α mimics some of the effects of AMPK activation provides strong support for an important role of PGC1α in mediating these effects.

## STAR★METHODS

Detailed methods are provided in the online version of this paper and include the following:

### RESOURCE AVAILABILITY

#### Lead contact

Further information and requests for resources and reagents should be directed to the lead contact, David Carling (dcarling@imperial.ac.uk).

#### Materials availability

All unique materials in this study are available upon request to the lead contact subject to a materials transfer agreement.

#### Data and code availability

- RNA-sequence datasets used in this study are available from Gene Expression Omnibus with the accession number GSE214601. Proteomic datasets are available from PRIDE (accession number PXD040731), and lipidomic datasets from MassIVE (MSV000091405).
- This paper does not report original code.
- Any additional information required to reanalyze the data reported in this paper is available from the lead contact upon request.

### EXPERIMENTAL MODEL AND SUBJECT DETAILS

#### Animal models

All *in vivo* studies were performed in accordance with the United Kingdom Animals (Scientific Procedures) Act (1986) and approved by the Animal Welfare and Ethical Review Board at Imperial College London. We made use of a genetic gain-of-function AMPK mouse model, harboring a mutation of aspartic acid residue 316 to alanine in Prkag1 (the γ1 subunit of AMPK) described elsewhere.^12^ The γ1 transgene contains a C-terminal FLAG epitope tag (DYKDDDDK). Male mice with prostate-specific deletion of *Pten* with and without prostate-specific activation of AMPK were generated by crossing female *Pten*^fl/fl^;*Ampk*γ*1*^fl/^ mice with male *Pbsn-cre4*^+^; *Pten*^fl/^;*Ampk*γ*1*^*fl*/^ transgenic mice. All experimental animals were maintained on a C57BL/6J genetic background and fed a chow-standard breeding diet number 3 (Special Diets Services) unless otherwise stated. For high-fat diet (HFD) feeding, HFD (60% energy from fat) was obtained from Ssniff (Soest, Germany).

### METHOD DETAILS

#### Histology

Whole prostates were excised and the bladder removed before weighing. Prostates were divided in half along the sagittal plane to give two equal samples comprising an anterior, dorsolateral and ventral lobe. One of the halves was immediately frozen in liquid nitrogen and used for subsequent studies (described below). The other half was fixed in 4% paraformaldehyde (PFA) overnight, wax embedded in paraffin, and sectioned to a thickness of 4 μm. Sections were stained with hematoxylin and eosin (H&E) and assessed for disease grading. Our pathology studies describe phenotypes of the dorsolateral prostate. Early adenocarcinoma is defined by areas of stromal micro-invasion. Advanced adenocarcinoma is defined by areas of extensive stromal invasion. For immunohistochemical staining, sections were deparaffinized and rehydrated in xylene (100%), followed by ethanol (70%), and boiled in sodium citrate antigen retrieval solution for 5 min in a pressure cooker. Sections were incubated with 0.3% (v/v) H_2_O_2_ to block endogenous peroxidase activity, washed with tris-buffered saline (TBS), and blocked for 1 h with 10% normal goat serum in TBS at room temperature. Sections were incubated overnight at 4°C with primary antibody (Ki-67 Abcam16667 (1:200); cleaved-caspase-3 CST#9661 (1:200); TOMM20 Abcam186735 (1:100); NF-κB CST#8242 (1:1000)). Sections were washed with TBS-Tween (0.1%) and incubated with biotinylated goat secondary antibody for 1 h at room temperature. Sections were then washed with TBS-Tween (0.1%) and incubated for 30 min with avidin–biotin complex (VECTASTAIN Elite ABC Kit, Vector Laboratories) according to manufacturer’s instructions. Sections were washed with TBS and stained using the DAB Substrate Kit (Vector Laboratories) according to the manufacturer’s instructions before counterstaining with Gill hematoxylin (Sigma). Sections were then dehydrated and mounted using DPX mountant (Sigma). For quantification using QuPath,^51^ automated cell detection using the color deconvolved haematoxylin stain was first performed within manually annotated epithelial cell regions. For NF-κB, following cell detection, the nucleus to cytoplasmic DAB mean optical density ratio was calculated per cell.

#### Organoid generation

Mouse prostate organoids from wild-type (WT), *Pten*^−/−^ and *Pten*^−/−^;*Ampk*^ACT^ mice were generated and cultured as previously described.^52^ However, collagenase/hyaluronidase (STEMCELL Technologies no. 07912) digestion at 37°C was reduced from 3 h to 2 h and E-cadherin PerCP-Efluor710 antibody (eBiosciences 46-3249-82) was substituted with E-cadherin PE antibody (Biolegend 14730) for sorting of prostate epithelial cells.

#### Cell culture

C4–2 and C4–2b cells were obtained from ATCC. Cells were cultured in RPMI 1640 medium, GlutaMAX (Gibco-61870), and supplemented with 10% FBS (Sigma-Aldrich), 100 U/mL penicillin, and 100 μg/mL streptomycin. All cells were maintained at 37°C and 5% CO_2_ and tested for Mycoplasma using MycoAlert. For all studies, passage number was kept below 25. In some cases, cells were treated with BI9774 (a kind gift from Dr. Jon Read, AstraZeneca) or etomoxir (Cayman Chemical). Specific conditions and concentrations are indicated in individual figure legends. For overexpression of PGC1α, pcDNA4mycPGC-1α ^50^ (Adgene #10974) was transiently transfected into C4–2 cells using Lipofectamine 3000 (Invitrogen) according to the manufacturer’s instructions. As a control, pLPC-N-myc (Adgene#1254) was transfected using the same conditions.

#### Western blotting

Organoids were harvested by centrifugation (340 × g) and washed in phosphate-buffered saline (PBS), before homogenization and sonification in RIPA buffer. C4–2 cells were washed briefly in PBS before lysis in RIPA buffer. Samples were then centrifuged at 13,000 × g for 15 min to remove insoluble material. Protein content of the soluble fraction was quantified using a BCA assay kit (ThermoScientific). Proteins were resolved by SDS-PAGE (Novex bis-tris 4%–12% gradient gels) and transferred to polyvinylidene difluoride membrane. Primary antibodies were used at 1:1000 dilution. The following antibodies were from Cell Signaling Technology: AMPKα1/2 (#2793), AMPKβ1/2 (#4150), ACC (#3676), pACC (#3661), pAMPKαThr172 (#2535), AKT (#2920), pAKTSer473 (#4060) and SNAI1 (#3879). Other antibodies used in this study are as follows: ACC (Millipore, 05–1098), FLAG (Sigma, F7425), PGC1α (Novus, NP1–04676), total OXPHOS antibody cocktail (Abcam, ab110413), FBP1 (Abcam, 109020) and Vinculin (Sigma, V9131). Primary antibodies were detected using LI-COR IRDye Infrared Dye- or HRP-conjugated secondary antibodies and visualized using an Odyssey Infrared Imager (LI-COR Biotechnology) or Amersham ImageQuant 800 system, respectively. Quantification of results was performed using Odyssey software (LI-COR) or ImageJ (ECL) and expressed as a ratio of the signal relative to the signal obtained using an appropriate control antibody.

#### Cell proliferation and invasion assays

Cell proliferation and invasion assays were performed on the xCELLigence RTCA DP (ACEA Biosciences, Inc.) and data analyzed using the RTCA 2.0 Software. For proliferation assays, E-plates (Cambridge BioScience) were used and performed according to manufacturer’s instructions (8000 cells were plated). For invasion assays, CIM-plates (Cambridge BioScience) were coated with Matrigel (BD Biosciences) using a 1:40 dilution in serum-free media. Invasion assays were performed according to the manufacturer’s instructions. 40,000 cells were plated and invaded down a serum gradient of 0%–10%. Assays were performed over 48 h with readings taken every 15 min.

#### Lipid dependent oxygen consumption using seahorse XF96 Extracellular Flux analyser

Organoids: Prostate organoids were passaged and allowed to grow in culture for 1 week and were visible by eye. 96-well Seahorse cell culture plates were coated with 15 μL per well of 1:10 dilution of Matrigel in Seahorse XF DMEM Medium supplemented with Seahorse XF Glucose (10 mM) and Seahorse XF L-Glutamine (2 mM), left at 37°C for 30 min and stored at 4°C for up to 1 week before use. On the day of the assay, Matrigel coated plates were brought to room temperature at least 30 min before organoid plating. Organoids were harvested via centrifugation and washed into assay medium minus palmitate-BSA or BSA (Seahorse XF DMEM Medium supplemented with Seahorse XF glucose (10 mM), Seahorse XF L-glutamine (2 mM), 2% charcoal-stripped FBS (Gibco #12676) and 10 μM ROCK inhibitor Y-27632 (STEMCELL Technologies #07171)). Before plating, the coated 96-well culture plate was washed and 15 μL assay medium minus palmitate-BSA or BSA added to each well to aid even plating of prostate organoids. A P200 pipette tip was used to pick up 1–2 organoids in a volume of 15 μL volume and organoids were then dispensed in the center of each well. Culture plates were centrifuged at 340 × g for 15 min with slow deceleration to bring organoids to the bottom of the well. Organoid loaded plates were then placed in 37°C incubator without CO_2_ for 30 min to allow organoid attachment. Finally, 120 μL of assay medium and 30 μL palmitate-BSA or BSA (Agilent 102720–100) was added to each well (final concentration 150 μM palmitate). For the assay, etomoxir (final concentration 100 μM) was added to injection port A and rotenone and antimycin A (final concentration 2 μM) to port B. Assay was run on the Seahorse XF96 Extracellular Flux analyser with 4 cycles run for each condition (mix 3 min, wait 0 min, measure 3 min). Data were collected and analyzed using Agilent Seahorse Wave software version 2.6.1. Each plate was imaged before and after Seahorse assay to confirm organoids were in the center of the well and did not move during the assay. Wells where organoids were not in the central region or that had moved during the assay were excluded. Results were normalized per well based on basal oxygen consumption rate (OCR).

C4–2 cells: 96-well Seahorse cell culture plates were coated with Poly-L-Lysine before cell plating. 20 000 cells were seeded per well the night before the assay in cell assay medium minus palmitate-BSA or BSA (Seahorse XF DMEM Medium supplemented with Seahorse XF glucose (10 mM), Seahorse XF L-glutamine (2 mM), 5% charcoal-stripped FBS (Gibco #12676)). One the day of the assay, cells were washed and assay medium with palmitate-BSA or BSA added to give a final palmitate concentration of 150 μM. For the assay, etomoxir (final concentration 50 μM) was added to injection port A and rotenone and antimycin A (final concentration 0.5 μM) to port B. Assay was run on the Seahorse XF96 Extracellular Flux analyser with 3 cycles run for each condition (mix 3 min, wait 0 min, measure 3 min). Data were collected and analyzed using Agilent Seahorse Wave software version 2.6.1. After the assay, cells were fixed with 4% PFA and quantified using crystal violet staining, based on absorbance at 590 nm after solubilization in 10% acetic acid to confirm even plating. Results were normalized per well based on basal OCR.

#### Determination of cellular ATP

ATP was measured using CellTiter-Glo 2.0 according to manufacturer’s instructions and normalized to cell number assessed by the alamarBlue cell viability assay. alamarBlue cell viability assays were carried out according to manufacturer’s instructions.

#### RNA sequencing

Immediately following dissection, whole prostates were weighed and then divided in half along the sagittal plane to give two equal samples comprising an anterior, dorsolateral and ventral lobe. One-half was snap frozen in liquid nitrogen, ground under liquid nitrogen using a pestle and mortar, and used for subsequent transcriptomic, proteomic and lipidomic analyses. RNA was isolated by homogenization in Trizol reagent (Invitrogen) according to the manufacturer’s instructions, followed by purification on an RNeasy column (Qiagen). RNA quality was assessed using the Agilent 2100 RNA 6000 Nano assay and libraries prepared using the NEBNext Ultra II Directional RNA Library Prep Kit with NEBNext Poly(A) mRNA Magnetic Isolation Module following manufacturer’s instructions. Library quality was evaluated using the Agilent 2100 High-Sensitivity DNA assay, and concentrations measured using the Qubit dsDNA HS Assay Kit. Libraries were sequenced on a NextSeq 500 to generate a minimum of 40 million Paired End 41bp reads per sample. Reads were aligned to Ensembl mouse genome (GRCm38) using TopHat2 version 2.1.1.^53^ Mapped reads that fell on genes were counted using featureCounts from Rsubread package.^54^ Generated count data were then used to normalise and identify differentially expressed genes using DESeq2^55^ and DEGs were defined with Benjamini-Hochberg adjusted p < 0.05. Gene Set Enrichment Analysis was performed using GSEA software^56^ on pre-ranked list generated by DESeq2.

#### Gene expression by RT-qPCR

RNA was isolated from human C4–2 cells using Trizol reagent (Invitrogen) according to the manufacturer’s instructions followed by purification on an RNeasy column (Qiagen). First-strand cDNA synthesis was performed using Superscript II (Invitrogen) followed by quantitative PCR using PowerUp SYBR Green according to manufacturer’s instructions. KiCqStart SYBR Green Primers were obtained from Sigma-Aldrich (Table S7). mRNA expression was normalized to house keeper (*UBC*) gene expression, the expression of which was unaltered between conditions.

#### Proteomic analysis

Protein was extracted from powdered prostate tissue (as described above) by homogenization in 8 M Urea and processed using an in-solution digestion procedure. Briefly, samples were sequentially reduced and alkylated at room temperature, in the dark for 30 min, with final concentrations of 10mM dithiothreitol (DTT) and 50mM 2-chloroacetamide respectively. Samples were then diluted 1:2 with 20mM HEPES (pH 8.0) for a final urea concentration of 4M. 200ng of LysC (Wako, 125–05061) was added and samples were incubated at 37°C for 4 h. This was followed by a further dilution to 1M urea with HEPES buffer and 2μg of trypsin gold (Promega, V5280) was added for an approximate 1:50 protease to protein ratio. Samples were then incubated at 37°C for 17 h. The digestion was stopped by acidification with trifluoroacetic acid (TFA) to a final concentration of 0.2% and protein digests were desalted using Glygen C18 spin tips (Glygen Corp, TT2C18.96). Tryptic peptides were eluted with 60% acetonitrile, 0.1% formic acid, dried by vacuum centrifugation and re-dissolved in 0.1% TFA. LC-MS/MS analysis was performed using an Ultimate 3000 RSLC nano liquid chromatography system (Thermo Scientific) coupled to a Q-Exactive mass spectrometer (Thermo Scientific) via an EASY spray source (Thermo Scientific). Eluted peptides were analyzed by the mass spectrometer operating in positive polarity using a data-dependent acquisition mode. Data were processed using the MaxQuant1 software platform (v1.6.2.3), with database searches carried out by the in-built Andromeda search engine against a Swissprot M. musculus database (version 20201111, number of entries: 17,056). In each case, lysates from 6 independent animals, aged 17 weeks, were analyzed in duplicate. Proteins with a fold change >1.2 and p < 0.05 were determined to be significantly different between genotypes.

#### Lipidomic analysis

Lipids were extracted from powdered prostate tissue using the Folch method.^57^ In brief, chloroform/methanol (2:1 (v/v), 1 mL) was added to approximately 30 mg powered tissue and thoroughly vortex mixed. LC-MS grade water (400 μL) was added and samples were vortex mixed for further 20 s and then centrifuged (13,200 × g, 15 min) to separate the organic and aqueous layers. The organic layer was isolated and dried down, before being reconstituted in chloroform/methanol (2:1 (v/v), 600 μL) and diluted 1:50 in isopropanol/acetonitrile/water (2:1:1 (v/v/v)). Lipid profiling was performed by liquid chromatography high-resolution mass spectrometry (LC-HRMS) using a Vanquish Flex Binary UHPLC system (Thermo Scientific) coupled to a Q-Exactive mass spectrometer (Thermo Scientific). Chromatographic separation was achieved using Acquity UPLC BEH C18 column (Waters, 50 × 2.1 mm, 1.7 μm) at 55°C and flow rate of 0.5 mL/min. Mobile phase A was acetonitrile: water 60:40 (v/v) and mobile phase B was isopropanol: acetonitrile 90:10 (v/v), both with either 10 mM ammonium acetate (negative ion mode), or 10 mM ammonium formate (positive ion mode) added. A gradient run was used, starting with 40% B, up to 99% B over 8 min, held at 99% B for 0.5 min and then returned to starting conditions. The heated electrospray ionization source (HESI) had parameters for positive/negative mode as follows: capillary voltage 3.5/2.5 kV, heater temperature 438°C, capillary temperature 320°C, S-lens RF level 50, sheath, auxiliary and sweep gas flow rate were 53, 14 and 1 unit, respectively. Lipidomics data acquisition was performed with Xcalibur software (version 4.1). Peak picking was performed after conversion to mzML format using XCMS,^58^ and peak areas normalised to an appropriate isotopically labeled internal standard and tissue weight. Lipid identification was performed by accurate mass using LIPID MAPS structure database (LMSD,^59^) and confirmed by MS/MS where possible.

#### C4–2b xenograft model

Male NOD scid gamma (NSG) mice (6–8 weeks of age) were purchased from Charles River (UK) and allowed to acclimatize for 1 week. Mice were inoculated by subcutaneous injection with 2.5 × 10^6^ C4–2b cells resuspended in 100 μL serum-free media supplemented with 100 μL Matrigel Basement Membrane Matrix High Concentration (Corning). Tumors were allowed to develop to a volume of around 90 mm^3^ before randomization into either Vehicle (5% (v/v) DMSO, 30% (w/v) sulphobutylether-β-cyclodextrin) or 30 mg/kg BI9774 (in Vehicle) groups. Treatments were administered via oral gavage once daily for 21 days. *In vivo* and *in vitro* drug metabolism and pharmacokinetics parameters for BI9774 are available on the opnMe website (https://opnme.com/molecules/ampk-bi-9774). Tumor size was measured every 7 days using digital calipers, and the volume was calculated using the formula: length*width*height*π/6.

#### Bioinformatic analysis

Publicly available data was downloaded from indicated Geo datasets. Analysis of TCGA data^25^ was performed using the family of R packages containing the TCGA data (https://rtcga.github.io/RTCGA). Disease Free Survival: Kaplan-Meier curves representing the disease-free survival (DFS) of patient groups selected according to the quartile expression of the gene(s) of interest. This type of analysis shows the differences in the relapse of the disease among the different subgroups of the population and is synonymous with biochemical recurrence. The inbuilt R function quantile was used to split the data in quartiles. Quartiles represent ranges of expression that divide the set of values into quarters. Quartile color code: Q1 (Blue), Q2 + Q3 (Green), Q4 (Red). Each curve represents the percentage (Y axis) of the population that exhibits recurrence of the disease along time (X axis, in months) for a given gene expression distribution quartile. Vertical ticks indicate censored patients. Coxph function in the package survival (version 3.4.0) was used to calculate p value and Hazard ratios using the Cox regression method. Correlation analysis: Plotted values correspond to the log2-normalized gene expression values (fluorescence intensity or RSEM-UQ) for *PPARGC1A* (X axis) and the expression score, calculated by the R package singscore, version 1.14.0^60^ for multiple genes of interest (Y axis), for each patient. Blue line represents linear regression, gray area indicates the limits of the confidence intervals and R and p indicate Pearson’s correlation coefficient and statistical significance, respectively. Data from GSE21034,^26^ GSE35988,^48^ GSE32269^49^ was downloaded and processed as required via Bioconductor and limma with linear model region and Bayesian statistics for differential gene expression. Data was log2 transformed and analyzed as *Z* score for relative expression. Data was visualised via BoxplotR. Survival analysis of GSE21024 was performed with IBM-SPSS statistics software.

### QUANTIFICATION AND STATISTICAL ANALYSIS

All data are presented as mean ± s.e.m. and subjected to statistical analysis using GraphPad Prism software. Immunoblots were analyzed either using Image Studio Light V5.2 (LI-COR Biosciences) or ImageJ software. Sample size was determined from our previously published work (Penfold et al. 2018). Statistical significance was determined using two-tailed unpaired Student’s t-test for one variable comparison between two groups. Fisher’s exact test was used to compare two groups on a dichotomous categorical outcome (for grading of tissue sections). For comparisons involving a single variable in three or more groups, one-way ANOVA with Tukey’s post-hoc test was performed. All data comparing two variables (e.g. genotype, diet) were analyzed by two-way ANOVA with uncorrected Fisher’s LSD post-hoc test. For gene expression data analysis in human PCa datasets, significance was tested using Kruksal-Wallis non-parametric test. For all analyses, significance was accepted at p < 0.05.

## Supplementary Material

1

2

3

4

5

## Figures and Tables

**KEY RESOURCES TABLE:** 

REAGENT or RESOURCE	SOURCE	IDENTIFIER

Antibodies		

ACC (Rabbit polyclonal)	Cell Signaling Technology	Cat#3661
ACC (Mouse monoclonal)	Millipore	Cat#05–1098; Clone 7D2.2
AKT (Pan) (Mouse monoclonal)	Cell Signaling Technology	Cat#2920; Clone 20D4
AMPK alpha 1/2 (Mouse monoclonal)	Cell Signaling Technology	Cat#2793; Clone F6
AMPK beta1/2 (Rabbit monoclonal)	Cell Signaling Technology	Cat#4150; Clone 57C12
Cleaved-caspase 3 (Rabbit polyclonal)	Cell Signaling Technology	Cat#9661
FBP1 (Rabbit monoclonal)	Abcam	Cat#109020; Clone EPR4619
FLAG (Rabbit polyclonal)	Sigma-Aldrich	Cat#F7425
Ki-67 (Rabbit monoclonal)	Abcam	Cat#ab16667; Clone SP6
NF-κB p65 (D14E12) (Rabbit monoclonal)	Cell Signaling Technology	Cat#8242
pAKTSer473 (Rabbit monoclonal)	Cell Signaling Technology	Cat#4060; Clone D9E
pAMPKαThr172 (Rabbit monoclonal)	Cell Signaling Technology	Cat#2535; Clone 40H9
PGC1a (Rabbit polyclonal)	Novus	Cat#NP1–04676
SNAI1 (Rabbit monoclonal)	Cell Signaling Technology	Cat#3879; Clone C15D3
TOMM20 (Rabbit monoclonal)	Abcam	Cat#ab186735; Clone EPR15581–54
Total OXPHOS antibody cocktail	Abcam	Cat#ab110413
Vinculin (Mouse monoclonal)	Sigma-Aldrich	Cat#V9131; Clone hVIN-1
Anti-mouse EpCAM APC	BioLegend	Cat#118214
Anti-mouse E-Cadherin PerCP-Efluor710	eBiosciences	Cat#46–3249–82
Goat Anti-Rabbit IgG, Biotinylated	Vector Laboratories	Cat#BA-1000–1.5
Goat Anti-Mouse IgG, HRP conjugate	Cell Signaling Technology	Cat#91196
Goat Anti-Rabbit IgG, HRP conjugate	Cell Signaling Technology	Cat#7074
IRDye 680RD Goat anti-Mouse IgG	LI-COR Biosciences	Cat#926–68070
IRDye 680RD Goat anti-Rabbit IgG	LI-COR Biosciences	Cat#926–68071
IRDye 800CW Goat anti-Mouse IgG	LI-COR Biosciences	Cat#926–32210
IRDye 800CW Goat anti-Rabbit IgG	LI-COR Biosciences	Cat#926–32211

Chemicals, peptides, and recombinant proteins		

Acetic acid	Sigma-Aldrich	Cat#A6283
Antimycin A	Sigma-Aldrich	Cat#A8674
BI9774	Dr. Jon Read, AstraZeneca	N/A
Chloroform	ThermoFisher	Cat#022920.K2
Collagenase/hyaluronidase solution (10x)	STEMCELL Technologies	Cat#07912
Crystal violet sln	Sigma-Aldrich	Cat#V5265
DHT	Sigma-Aldrich	Cat#A8380
Dispase	STEMCELL Technologies	Cat#07913
DMEM/F-12	Gibco	Cat#10565
DNaseI	STEMCELL Technologies	Cat#07900
DPX mountant	VWR	Cat#06522
Etomoxir	Cambridge Bioscience	11969–10mg-CAY
EquiSPLASH^™^ LIPIDOMIX^®^ Quantitative MS Internal Standard	Avanti Polar Lipids	Cat#330731
FBS	Sigma-Aldrich	Cat#F9665
FBS (Charcoal stripped)	Gibco	Cat#12676
Gill Hematoxylin	Sigma-Aldrich	Cat#GHS132–1L
Glutamax (100x)	Gibco	Cat#35050
Glygen C18 spin tips	Glygen Corp	Cat#TT2C18.96
HBSS	STEMCELL Technologies	Cat#37150
Hydrogen peroxide	Sigma-Aldrich	Cat#H1009
Lipofectamine 3000	ThermoFisher	Cat#L3000015
LysC	Wako	Cat#125–05061
Matrigel (Organoid culture)	Corning	Cat#354234
Matrigel (Xenografts)	Corning	354248
NEBNext Ultra II Directional RNA Library Prep Kit	NEB	Cat#E7760
NEBNext Poly(A) mRNA Magnetic Isolation Module	NEB	Cat#E7490
Normal goat serum	ThermoFisher	Cat#16–210–064
Penicillin-Streptomycin	ThermoFisher	Cat#15140122
Paraformaldehyde	Sigma-Aldrich	Cat#158127
Poly-L-lysine	Sigma-Aldrich	Cat#P4707
Qubit^™^ dsDNA HS Assay Kit	ThermoFisher	Cat#Q32851
RIPA buffer	ThermoFisher	Cat#89900
ROCK inhibitor Y-27632	STEMCELL Technologies	Cat#07171
Rotenone	Sigma-Aldrich	Cat#R8875
RPMI-1640	Gibco	Cat#61870036
Sulphobutylether-β-cyclodextrin	MedChemExpress	Cat#HY-17031
TRIzol reagent	ThermoFisher	Cat#15596018
Trypsin/EDTA	STEMCELL Technologies	Cat#07901
Trypsin Gold	Promega	Cat#V5280
Tween 20	VWR	Cat#0777–1L
Xylene	Sigma-Aldrich	Cat#534056–4L

Critical commercial assays		

alamarBlue Cell Viability Reagent	ThermoFisher	Cat#DAL1100
BCA Protein Assay	ThermoFisher	Cat#23225
CellTiter Glo 2.0 Cell Viability	Promega	Cat#G9242
DAB Substrate Kit	Vector Laboratories	Cat#SK-4100
VECTASTAIN ELITE ABC-HRP KIT	Vector Laboratories	Cat#PK-6100
PowerUp SYBR Green Master Mix	Life Technologies	Cat#A25742
RNeasy Mini Kit Qiagen	Qiagen	Cat#74106
Seahorse FluxPaks	Agilent	Cat#102416–100
Seahorse XF DMEM Medium pH7.4	Agilent	Cat#103575–100
Seahorse XF 1.0m Glucose Solution	Agilent	Cat#103577–100
Seahorse XF 200 mm glutamine solution	Agilent	Cat#103579–100
Seahorse XF Palmitate-BSA FAO Substrate Kit	Agilent	Cat#102720–100
SuperScript II Reverse Transcriptase	ThermoFisher	Cat#18064014
RTCA CIM-plates (migration)	Agilent	Cat#5665825001
RTCA E-plates (proliferation)	Agilent	Cat#5469813001

Deposited data		

Lipidomics datasets	This study	MassIVE: MSV000091405
Proteomics datasets	This study	PRIDE: PXD040731
RNAsequencing	This study	GEO: GSE214601
TCGA PRAD dataset	Cancer Genome Atlas Research Network^25^	N/A
Primary and metastatic PCa	Taylor et al.^26^	GEO: GSE21034
CRPC dataset	Grasso et al.^48^	GEO: GSE35988
Primary and metastatic PCa	Cai et al.^49^	GEO: GSE32269

Experimental models: Cell lines		

C4–2	ATCC	Cat#CRL-3314
C4–2b	Prof. lan Mills, Oxford	N/A

Experimental models: Organisms/strains		

Mouse: Male C57BL/6J Pbsn-cre4+; Pten; Ampkg1(D316A)	This study	N/A
Mouse: Male C57BL/6J Pbsn-cre4+; Pten; Ampkg1(WT)	This study	N/A
Mouse: Male NOD.Cg-Prkdcscid Il2rgtm1Wjl/SzJ (NSG)	Charles River (UK)	Cat#005557

Oligonucleotides		

Primers for qPCR. See Table S7.	Sigma-Aldrich	N/A

Recombinant DNA		

pcDNA4 myc PGC-1 alpha	Addgene (Deposited by Toren Finkel)^50^	Cat#10974
pLPC N-terminal myc tag	Addgene (Deposited by Titia de Lange)	Cat#12540

Software and algorithms		

Agilent Seahorse Wave 2.6.1	Agilent	https://www.agilent.com/
CFX manager	BioRad	https://www.bio-rad.com/
Fiji/ImageJ	NIH	https://imagej.net; RRID: SCR_003070
GraphPad Prism 9	GraphPad Software	https://www.graphpad.com
Image Studio Lite V5.2	LI-COR Biosciences	https://www.licor.com/
LIPID MAPS^®^ structure database	https://doi.org/10.1093/nar/gkl838	https://www.lipidmaps.org/
QuPath	QuPath^51^	https://qupath.github.io/
RTCA Pro Software 2.6.0	Agilent	https://www.agilent.com/
Xcalibur software	ThermoFisher	https://www.thermofisher.com/order/catalog/product/OPTON-30965
XCMS	Bioconductor	https://bioconductor.org/packages/release/bioc/html/xcms.html
Zen 2.3 Lite	Zeiss	https://www.zeiss.com/microscopy/

Other		

Acquity UPLC BEH C18 column	Waters	Cat#186002350
Immobilon-P PVDF membrane	Millipore	Cat#IPVH00010
Mouse chow diet RM3 diet	LBS Biotech	Cat#1011037
Mouse high fat diet (60%)	Ssniff (Soest, Germany)	Cat#E15742–347 (D12492)
NuPage Bis-Tris gradient gels (4–12%)	ThermoFisher	Cat#NP0336BOX

## Highlights

- Chronic genetic activation of AMPK protects against prostate cancer *in vivo*
- AMPK activation induces PGC1α and catabolic reprogramming of prostate cancer cells
- The catabolic state correlates with inhibition of a network of cell cycle genes
- AMPK activation decreases storage of neutral lipids in prostate cancer cells
