# Supplementary material for: AMPK activation protects against prostate cancer by inducing a catabolic cellular state
Source: Cell Rep. Author manuscript; Available in PMC 2023 Oct 23. (PMC10576838; doi:10.1016/j.celrep.2023.112396)
Supplement: 1 [file NIHMS1897945-supplement-1.pdf]

Cell Reports, Volume *42*

Supplemental information

AMPK activation protects against prostate cancer by inducing a catabolic cellular state

Lucy Penfold, Angela Woods, Alice E. Pollard, Julia Arizanova, Eneko Pascual-Navarro, Phillip J. Muckett, Marian H. Dore, Alex Montoya, Chad Whilding, Louise Fets, Joao Mokochinski, Theodora A. Constantin, Anabel Varela-Carver, Damien A. Leach, Charlotte L. Bevan, Alexander Yu. Nikitin, Zoe Hall, and David Carling

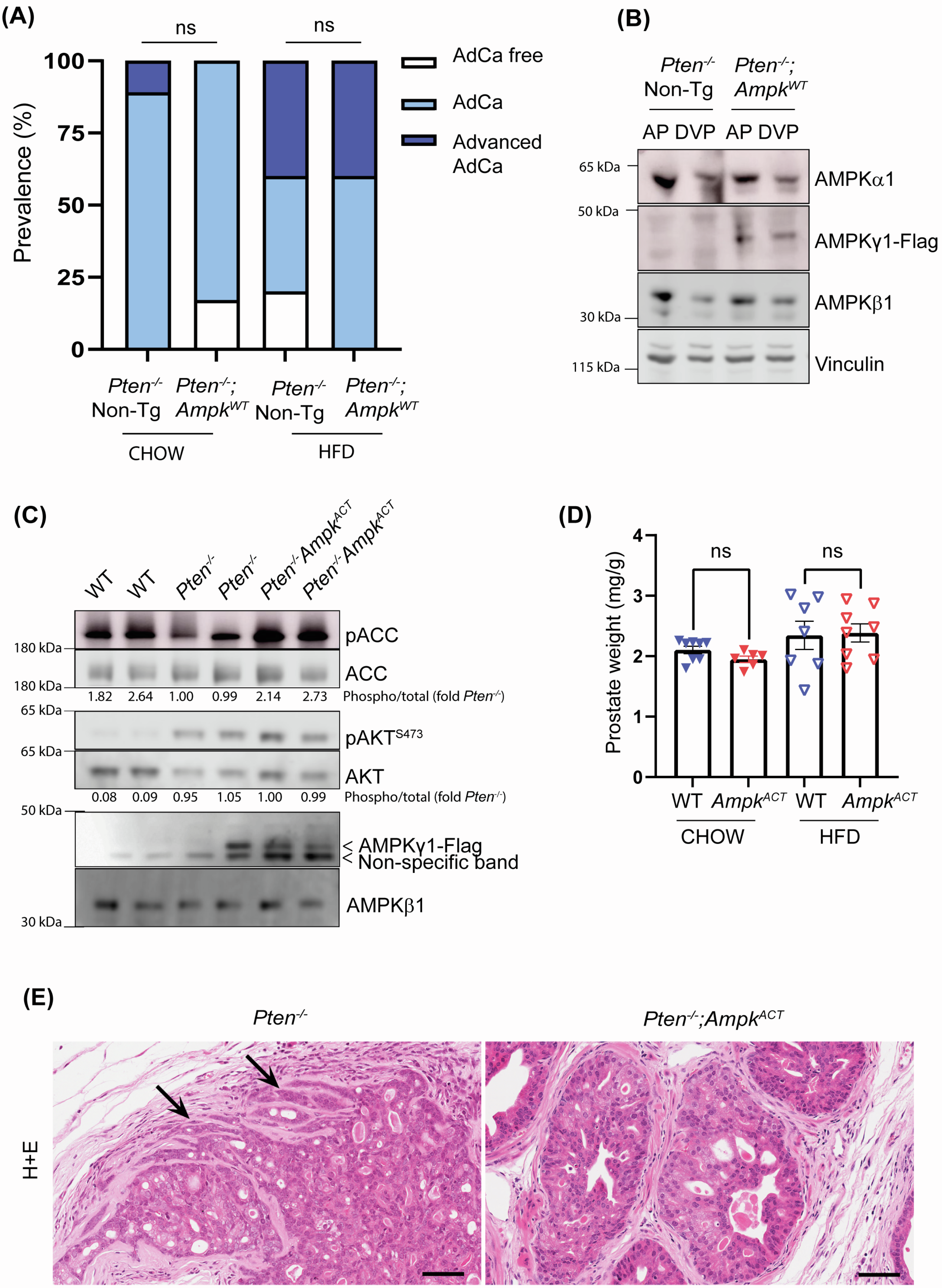
(A)
ns
ns
100
75
50
25
0
Prevalence (%)
AdCa free
AdCa
Advanced AdCa
Pten-/- Non-Tg
Pten-/-; AmpkWT
Pten-/- Non-Tg
Pten-/-; AmpkWT
CHOW
HFD
(B)
Pten-/- Non-Tg
Pten-/-; AmpkWT
AP DVP AP DVP
65 kDa
50 kDa
30 kDa
115 kDa
AMPKα1
AMPKγ1-Flag
AMPKβ1
Vinculin
(C)
WT
WT
Pten-/-
Pten-/-
Pten-/- AmpkACT
Pten-/- AmpkACT
180 kDa
180 kDa
pACC
ACC
1.82 2.64 1.00 0.99 2.14 2.73
Phospho/total (fold Pten-/-)
65 kDa
65 kDa
pAKTS473
AKT
0.08 0.09 0.95 1.05 1.00 0.99
Phospho/total (fold Pten-/-)
50 kDa
30 kDa
AMPKγ1-Flag
Non-specific band
AMPKβ1
(D)
ns
ns
4
3
2
1
0
Prostate weight (mg/g)
WT AmpkACT WT AmpkACT
CHOW
HFD
(E)
Pten-/-
Pten-/-;AmpkACT
H+E

Figure S1. Expression of wild-type *Prkag1* has no effect on PCa disease progression. Related to Figure 1.

(A) Quantification of pathological grading from H&E-stained sections isolated from mice aged 17 weeks maintained on chow diet and mice aged 52 weeks that had been fed a high fat diet (HFD) for the final 12 weeks. All mice lacked *Pten* expression in the prostate (*Pten*$^{-/-}$) and were either positive (*Ampk*^{WT}) or negative (Non-Tg) for prostate-specific expression of wild-type *Prkag1* transgene. Data shown are for n=9 (*Pten*$^{-/-}$, Chow), n=6 (*Pten*$^{-/-}$;*Ampk*^{WT}, Chow), n=5 *Pten*$^{-/-}$, HFD), and n=5 (*Pten*$^{-/-}$;*Ampk*^{WT}, HFD). AdCa; adenocarinoma. (B) Western blot analysis of endogenous AMPK α1 and β1 subunits in anterior prostate (AP) and dorsolateral ventral prostate (DVP) lobes isolated from Non-Tg or *Ampk*^{WT} *Pten*$^{-/-}$ mice. Expression of the *Ampk*^{WT} transgene was detected using an anti-FLAG antibody. Vinculin was used to monitor total protein levels. (C) Western blot analysis of prostate organoids generated from wild-type (WT), *Pten*$^{-/-}$, *Pten*$^{-/-}$;*Ampk*^{WT} and *Pten*$^{-/-}$;*Ampk*^{ACT} mice aged 1 year. Quantification is shown beneath respective blots and represents level of phosphorylated/total protein (fold *Pten*$^{-/-}$). Note that one of the *Pten*$^{-/-}$ organoid cultures expresses Flag-tagged wild-type γ1. (D) Weight of whole prostate isolated from wild type (WT) mice or *Ampk*^{ACT} mice at ~14 weeks of age fed a chow diet or from mice aged ~36 weeks of age that had been fed a HFD diet for 16 weeks (from ~20 weeks of age). (E) Representative H&E-stained prostate sections collected from *Pten*$^{-/-}$ or *Pten*$^{-/-}$;*Ampk*^{ACT} mice after 12 weeks HFD diet are shown and illustrate advanced AdCa and PIN4, respectively. Areas of stromal invasion by AdCa are shown by arrows on *Pten*$^{-/-}$ section. Scale bar: 60 µm.

(A)

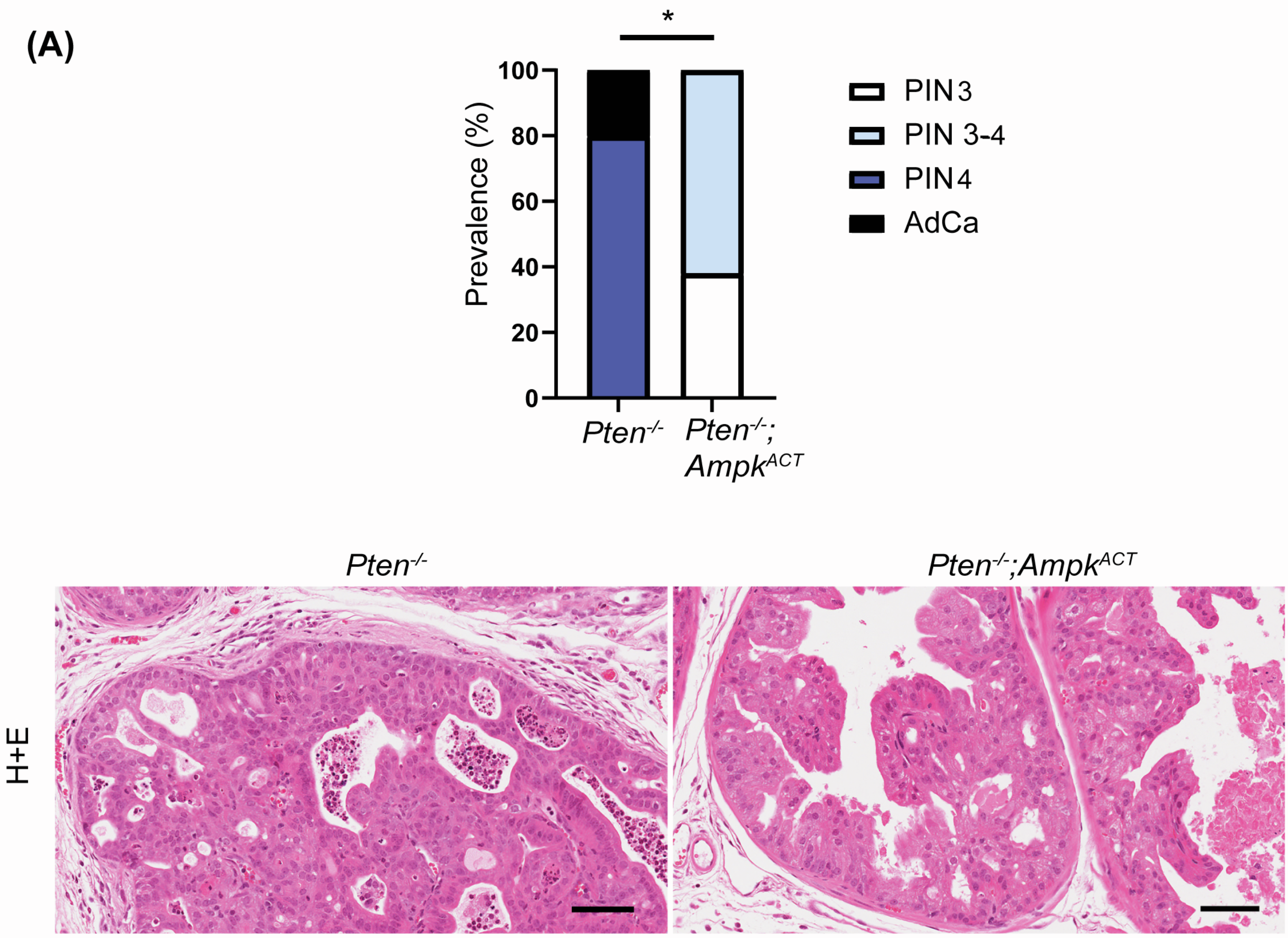
*
100
80
60
40
20
0
Prevalence (%)
PIN3
PIN 3-4
PIN4
AdCa
Pten-/-
Pten-/-;
AmpkACT
Pten-/-
Pten-/-;AmpkACT
H+E

(B)

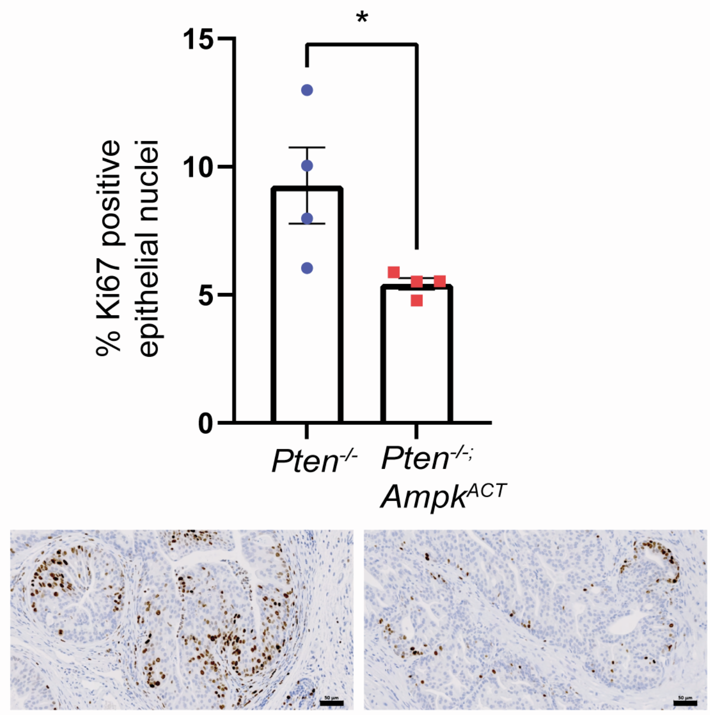
*
15
10
5
0
% Ki67 positive
epithelial nuclei
Pten-/-
Pten-/-;
AmpkACT

Figure S2. Characterisation *Pten*$^{-/-}$ and *Pten*$^{-/-}$*;Ampk*^{ACT} mice at 17 weeks. Related to Figure 1.

(A) Quantification of grading using H&E-stained sections from *Pten*$^{-/-}$ or *Pten*$^{-/-}$;*Ampk*^{ACT} mice aged 17 weeks. AdCa; adenocarinoma. Significant difference between AdCa and PIN4 groups vs all other groups is shown using Fisher exact test (two-sided p value) *$p < 0.05$, (n=5 for *Pten*$^{-/-}$; n=8 for *Pten*$^{-/-}$;*Ampk*^{ACT}). Representative H&E-stained prostate sections isolated from *Pte*n$^{-/-}$ or *Pten*$^{-/-}$;*Ampk*^{ACT} mice are shown and illustrate PIN4 and PIN3, respectively. Scale bar: 60 µm. (B) Percentage of Ki-67–positive cells in prostate epithelium of 17 week old mice (n=4 per genotype; at least 10,000 epithelial cells per section). Representative images of Ki-67–stained sections for each genotype are shown (scale bar: 50 µm). Data shown as mean ±SEM. Statistically significant differences between genotypes are shown as *$p < 0.05$.

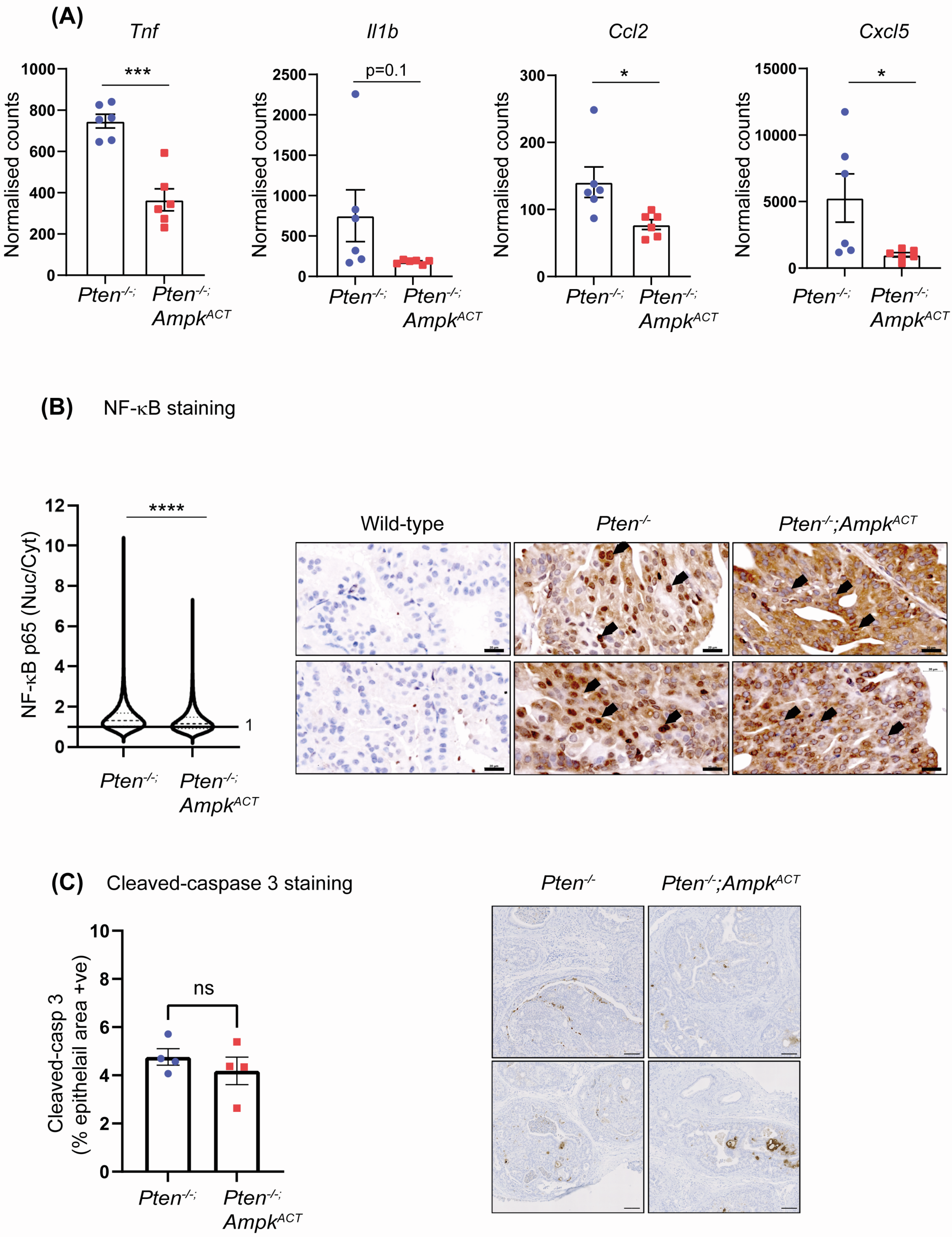
(A)
Tnf
Il1b
Ccl2
Cxcl5
Normalised counts
***
p=0.1
*
*
1000
800
600
400
200
0
2500
2000
1500
1000
500
0
300
200
100
0
15000
10000
5000
0
Pten-/-;
Pten-/-; AmpkACT
(B) NF-κB staining
NF-κB p65 (Nuc/Cyt)
****
12
10
8
6
4
2
0
1
Wild-type
Pten-/-
Pten-/-;AmpkACT
(C) Cleaved-caspase 3 staining
Cleaved-casp 3 (% epithelail area +ve)
ns
10
8
6
4
2
0

Figure S3. AMPK activation inhibits pro-inflammatory signalling in PCa. Related to Figure 2. (A) Gene expression of candidate pro-inflammatory genes is shown as determined by RNA-sequencing. Data shown as mean ±SEM (n=6 mice per genotype); Student's t-test was used to determine significant differences between genotypes *p <0.05, ***p <0.005. (B) Ratio of nuclear to cytosolic NF-κB staining and (C) quantification of cleaved caspase-3 staining, in prostate sections from wild-type (WT), *Pten*$^{-/-}$ and *Pten*$^{-/-}$;*Ampk*^{ACT} mice aged 17 weeks. In both cases, representative images are shown alongside the graphs (panel B, scale bar: 20 μm; panel C, scale bar: 100 μm.).

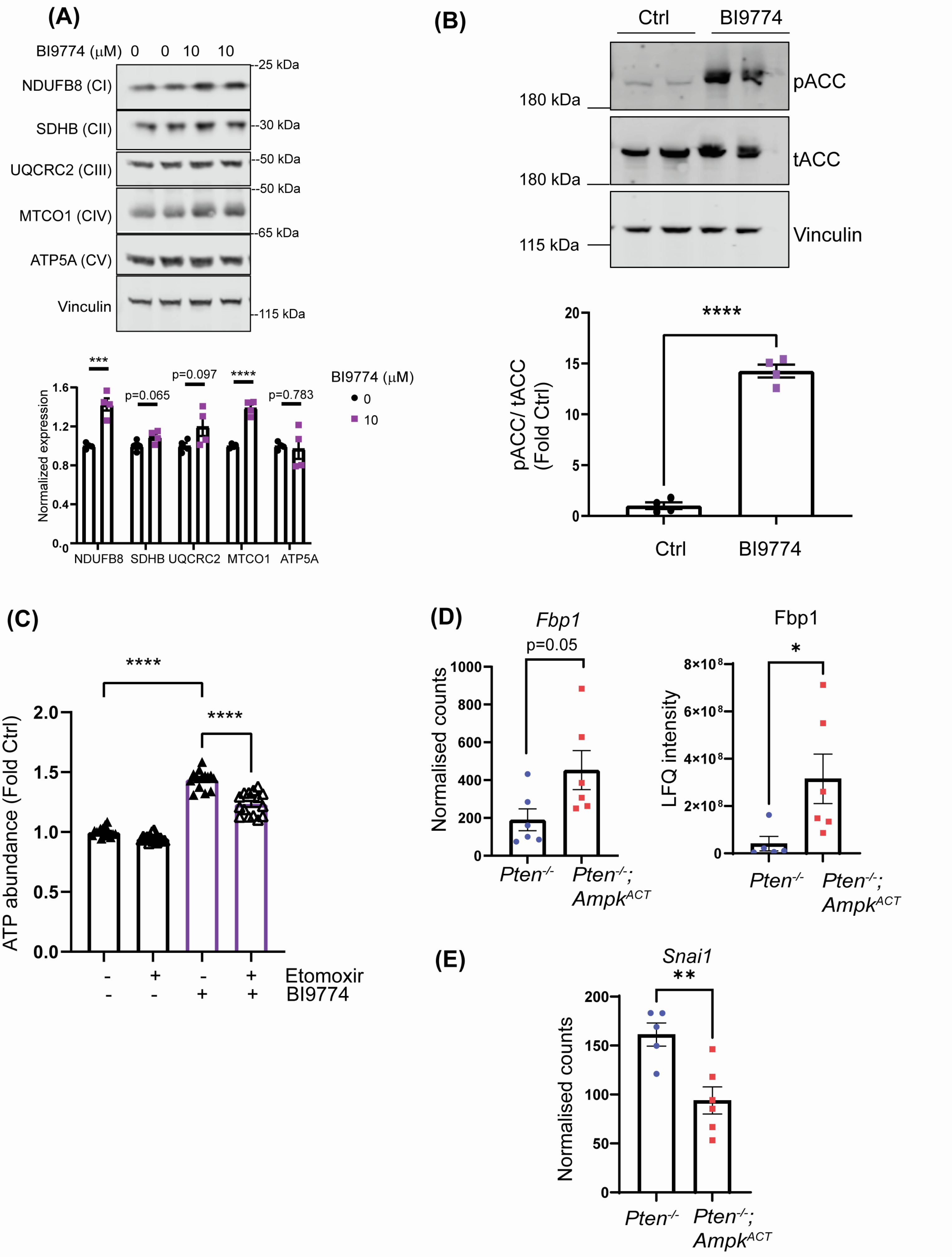
(A)
BI9774 (μM) 0 0 10 10
NDUFB8 (CI)
SDHB (CII)
UQCRC2 (CIII)
MTCO1 (CIV)
ATP5A (CV)
Vinculin
25 kDa
30 kDa
50 kDa
50 kDa
65 kDa
115 kDa
Normalized expression
NDUFB8 SDHB UQCRC2 MTCO1 ATP5A
***
p=0.065
p=0.097
****
p=0.783
BI9774 (μM)
0
10
(B)
Ctrl
BI9774
pACC
tACC
Vinculin
180 kDa
180 kDa
115 kDa
pACC/ tACC (Fold Ctrl)
****
Ctrl
BI9774
(C)
ATP abundance (Fold Ctrl)
****
****
Etomoxir
BI9774
(D)
Fbp1
p=0.05
Normalised counts
Pten-/-
Pten-/-; AmpkACT
Fbp1
*
LFQ intensity
Pten-/-
Pten-/-; AmpkACT
(E)
Snai1
**
Normalised counts
Pten-/-
Pten-/-; AmpkACT

Figure S4. Characterisation of C4-2 cells treated with BI9774 for 7 days. Related to Figure 4. **(**A) Representative Western blot analysis of mitochondrial electron transport chain proteins and quantification of normalized protein expression in cells treated in the presence or absence of 10 μM BI9774 for 7 days. (B) Quantification of ACC phosphorylation (pACC) relative to total ACC (tACC) protein, together with a representative Western blot, in cells treated as in (A). For (A) and (B) data shown are mean ±SEM (n=4 per condition) and Student's t-test was used to determine significant differences between groups *p <0.05, ***p <0.005. (C) ATP abundance was determined in cells treated ± BI9774 (10 μM) for 7 days, and treated ± etomoxir (50 μM) over the final 24 h. Data shown are means ±SEM (n=15 per condition) and one-way Anova with Tukey's post hoc test was used to determine significant differences between groups, ****p <0.001. (D) *Fbp1* mRNA and protein expression and (E) *Snai1* mRNA expression in $Pten^{-/-}$ and $Pten^{-/-}$;$Ampk^{ACT}$ mouse prostates from 17-week old mice. Data shown as mean ±SEM (n=6 per genotype); Student's t-test was used to determine statistically significant differences between genotypes *p <0.05, **p <0.01.

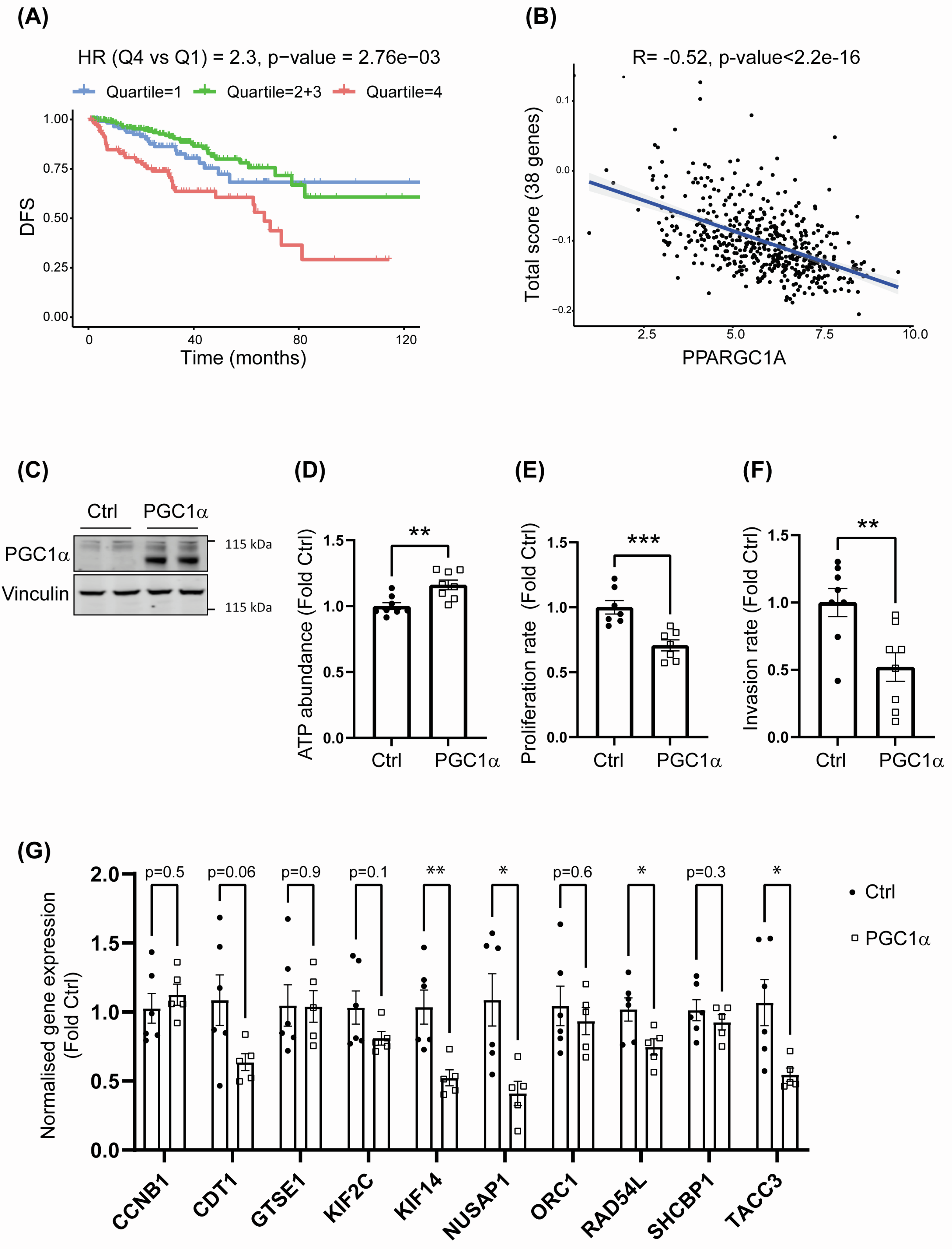

(A)
HR (Q4 vs Q1) = 2.3, p−value = 2.76e−03
Quartile=1
Quartile=2+3
Quartile=4
DFS
1.00
0.75
0.50
0.25
0.00
0
40
80
120
Time (months)
(B)
R= -0.52, p-value<2.2e-16
Total score (38 genes)
0.1
0.0
−0.1
−0.2
2.5
5.0
7.5
10.0
PPARGC1A
(C)
Ctrl
PGC1α
PGC1α
Vinculin
115 kDa
115 kDa
(D)
**
ATP abundance (Fold Ctrl)
1.5
1.0
0.5
0.0
Ctrl
PGC1α
(E)
***
Proliferation rate (Fold Ctrl)
1.5
1.0
0.5
0.0
Ctrl
PGC1α
(F)
**
Invasion rate (Fold Ctrl)
1.5
1.0
0.5
0.0
Ctrl
PGC1α
(G)
Normalised gene expression (Fold Ctrl)
2.0
1.5
1.0
0.5
0.0
p=0.5
p=0.06
p=0.9
p=0.1
**
*
p=0.6
*
p=0.3
*
Ctrl
PGC1α
CCNB1
CDT1
GTSE1
KIF2C
KIF14
NUSAP1
ORC1
RAD54L
SHCBP1
TACC3

Figure S5. Role of PGC1α in mediating the downstream effects of AMPK activation. Related to Figures 4 and 5.

(A) Gene expression of 38 genes identified as a cell cycle network in the significantly differentially expressed genes in prostates from 17 week old *Pten*$^{-/-}$ and *Pten*$^{-/-}$;*Ampk*^{ACT} mice in human PCa separated by disease free survival (DFS) using the TCGA PRAD dataset [25] analysed using CANCERTOOL. Inset shows tabulated data for Hazard ratio (HR) between Q4 and Q1 and p-value. (B) Correlation of gene expression with *PGC1α* (*PPARGC1A*) gene expression using the TCGA PRAD dataset analysed using CANCERTOOL. Inset shows tabulated data for Rank Correlation Coefficient (R) value and p-value. (C) C4-2 cells were transiently transfected with PGC1α or a control. 72 hours post-transfection, PGC1α expression was determined by Western blot analysis. (D) ATP abundance, (E) cell proliferation and (F) cell invasion were determined (mean ±SEM, n=7-8). (G) mRNA expression for the 10 “AMPK-Cell Cycle” genes was determined (mean ±SEM, n=4-5). Student’s t-test was used to determine significant differences between groups *p <0.05, **p <0.01, ***p < 0.005.

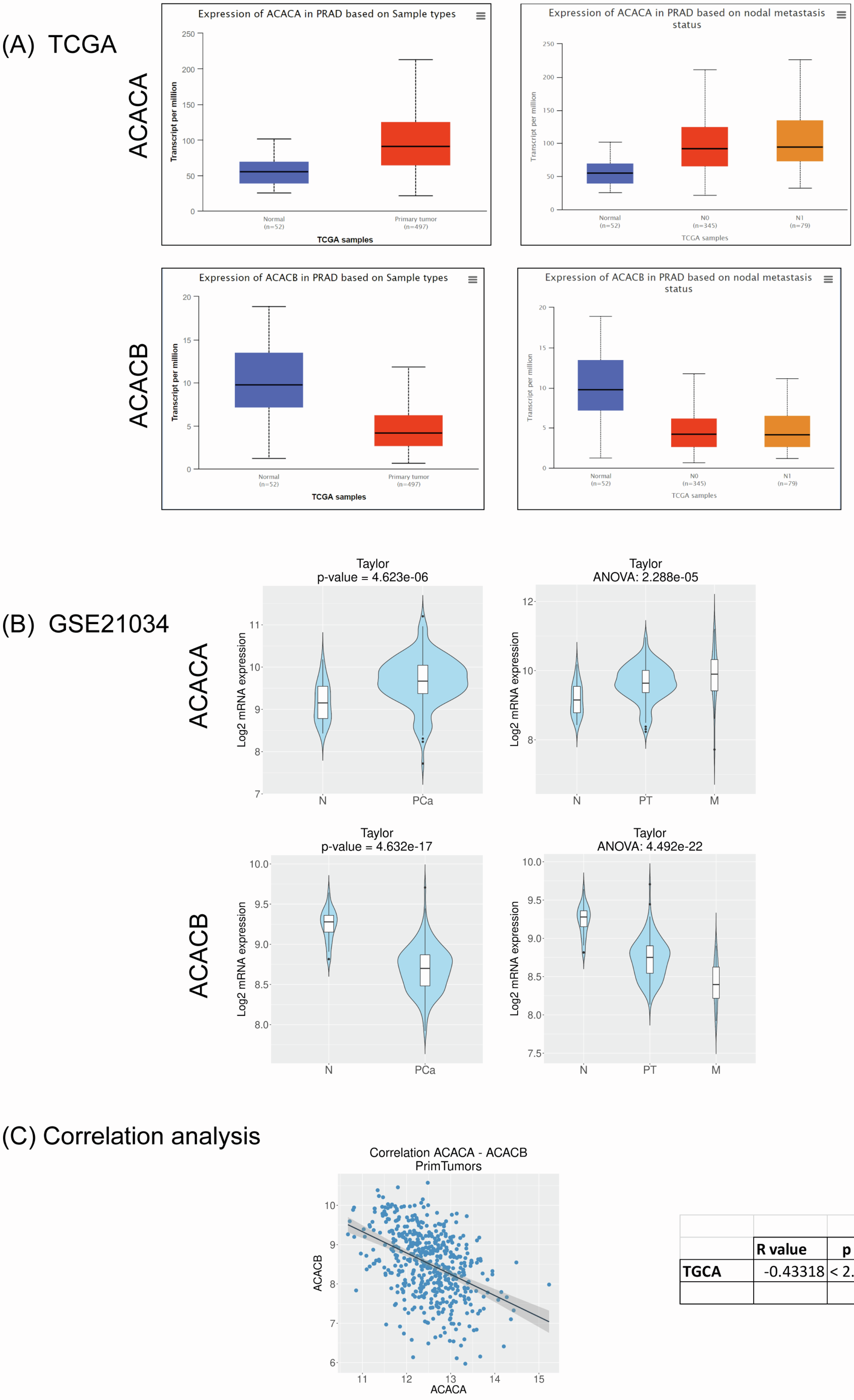

	**R value**	**p value**
**TGCA**	-0.43318	< 2.2e-16

Figure S6. *ACACA* and *ACACB* gene expression in human PCa. Related to Figures 3 and 6.
(A) *ACACA* and *ACACB* gene expression in human prostate separated by disease status (normal, no nodal PCa metastasis, or with nodal PCa metastasis), using the TCGA PRAD dataset [25] (n=497), analysed using UALCAN. (B) *ACACA* and *ACACB* gene expression in human prostate separated by disease status (N, normal; PT, primary tumour; M, metastasis) using the GSE21034 dataset [26] (benign n=29, localised cancer n=131, metastasis n=19). (C) Correlation of ACACA and ACACB gene expression in human PCa using the TCGA PRAD dataset. Inset shows tabulated data for Rank Correlation Coefficient (R) value and p-value. Data in (B) and (C) analysed using CANCERTOOL.

Table S1. List of genes identified as *PPARGC1A* targets from Torrano et al. [22]. Related to Figures 2 and 3.

Gene symbol	adj. p value	p Value	log2FC	FC	Gene name	Identified in proteomic analysis
ECM1	2.61E-05	1.46E-09	2.528707	5.770541	extracellular matrix protein 1	Yes
ATP1B1	0.000785	2.19E-07	1.61885	3.071301	ATPase Na+/K+ transporting subunit beta 1	Yes
MPC1	0.002599	1.23E-06	1.175036	2.257985	mitochondrial pyruvate carrier 1	Yes
SPINK13	0.044286	4.30E-04	1.120446	2.174142	serine peptidase inhibitor, Kazal type 13 (putative)	No
GOT1	0.00044	7.91E-08	1.022392	2.031284	glutamic-oxaloacetic transaminase 1	Yes
TP53INP2	0.007772	1.30E-05	0.961227	1.946965	tumor protein p53 inducible nuclear protein 2	No
CPLX1	0.002932	1.96E-06	0.944219	1.924147	complexin 1	No
UBC	0.002932	1.89E-06	0.871946	1.83013	ubiquitin C	Yes
GSTM1	0.005469	5.09E-06	0.824334	1.770717	glutathione S-transferase mu 1	Yes
ENDOG	0.002599	1.30E-06	0.823681	1.769916	endonuclease G	Yes
NDUFA4	0.001381	4.62E-07	0.816954	1.761683	NDUFA4, mitochondrial complex associated	Yes
ZDHHC23	0.008967	2.27E-05	0.798716	1.739552	zinc finger DHHC-type containing 23	No
COX7B	0.008153	1.77E-05	0.787894	1.726553	cytochrome c oxidase subunit 7B	No
IDH3A	0.010874	3.34E-05	0.781632	1.719075	isocitrate dehydrogenase 3 (NAD(+)) alpha	Yes
DECR1	0.005469	5.19E-06	0.769427	1.704592	2,4-dienoyl-CoA reductase 1, mitochondrial	Yes
WIPI1	0.031288	2.37E-04	0.75735	1.690383	WD repeat domain, phosphoinositide interacting 1	Yes
UQCRHL	0.011911	4.19E-05	0.752631	1.684862	ubiquinol-cytochrome c reductase hinge protein like	No
CLYBL	0.008485	1.94E-05	0.74501	1.675986	citrate lyase beta like	Yes
RNASE4	0.01299	4.64E-05	0.741299	1.67168	ribonuclease A family member 4	Yes
LDHD	0.031288	2.28E-04	0.737828	1.667664	lactate dehydrogenase D	Yes
MCCC1	0.006176	6.41E-06	0.704154	1.629189	methylcrotonoyl-CoA carboxylase 1	Yes
RBM47	0.039634	3.27E-04	0.68229	1.604685	RNA binding motif protein 47	Yes
COMTD1	0.014337	5.60E-05	0.67258	1.593921	catechol-O-methyltransferase domain containing 1	No
FASTKD1	0.005161	4.32E-06	0.670317	1.591423	FAST kinase domains 1	Yes
RNF14	0.006426	7.53E-06	0.669331	1.590335	ring finger protein 14	Yes
NDUFB9	0.006974	8.95E-06	0.647505	1.566456	NADH:ubiquinone oxidoreductase subunit B9	Yes
UQCRB	0.03171	2.46E-04	0.647012	1.565922	ubiquinol-cytochrome c reductase binding protein	Yes
FAHD1	0.007087	1.04E-05	0.642023	1.560516	fumarylacetoacetate hydrolase domain containing 1	Yes
MARC2	0.010462	3.15E-05	0.631896	1.549601	mitochondrial amidoxime reducing component 2	No
CYCS	0.006426	7.43E-06	0.631739	1.549431	cytochrome c, somatic	Yes
ARHGEF6	0.010266	2.92E-05	0.624106	1.541255	Rac/Cdc42 guanine nucleotide exchange factor 6	Yes
ATP5C1	0.022367	1.25E-04	0.615961	1.532579	ATP synthase, H+ transporting, mitochondrial F1 complex, gamma polypeptide 1	Yes
GPR3	0.014373	6.12E-05	0.612868	1.529296	G protein-coupled receptor 3	No
UQCRC2	0.014373	6.24E-05	0.609512	1.525743	ubiquinol-cytochrome c reductase core protein II	Yes
MTFR1L	0.010293	3.02E-05	0.604477	1.520428	mitochondrial fission regulator 1 like	Yes
MPC2	0.007087	1.11E-05	0.593638	1.509048	mitochondrial pyruvate carrier 2	Yes
FH	0.031288	2.27E-04	0.592707	1.508074	fumarate hydratase	Yes
PGAM4	0.010264	2.83E-05	0.590339	1.5056	phosphoglycerate mutase family member 4	No
KRTAP20-2	0.026391	1.68E-04	0.589596	1.504826	keratin associated protein 20-2	No
PRADC1	0.021482	1.18E-04	0.585724	1.500792	protease associated domain containing 1	No
NDUFB10	0.006974	8.63E-06	0.582636	1.497583	NADH:ubiquinone oxidoreductase subunit B10	Yes
MGST3	0.010264	2.86E-05	0.5785	1.493296	microsomal glutathione S-transferase 3	Yes
IMMT	0.026219	1.65E-04	0.575596	1.490293	inner membrane mitochondrial protein	Yes
GAS2L1	0.015068	6.89E-05	0.575317	1.490005	growth arrest specific 2 like 1	No
HIGD1A	0.023571	1.38E-04	0.571711	1.486285	HIG1 hypoxia inducible domain family member 1A	Yes
PPIC	0.01599	7.49E-05	0.571269	1.48583	peptidylprolyl isomerase C	Yes
ACO2	0.00862	2.02E-05	0.570878	1.485427	aconitase 2	Yes
CKB	0.014373	6.14E-05	0.55844	1.472676	creatine kinase B	Yes
DLD	0.030104	2.10E-04	0.550235	1.464324	dihydrolipoamide dehydrogenase	Yes
ACACB	0.026219	1.63E-04	0.546196	1.460231	acetyl-CoA carboxylase beta	No
ACADM	0.035188	2.85E-04	0.545046	1.459067	acyl-CoA dehydrogenase, C-4 to C-12 straight chain	Yes
PDHA1	0.007087	1.04E-05	0.544194	1.458206	pyruvate dehydrogenase (lipoamide) alpha 1	Yes
NNT	0.008963	2.15E-05	0.543037	1.457037	nicotinamide nucleotide transhydrogenase	No
ATP5H	0.007874	1.58E-05	0.540702	1.454681	ATP synthase, H+ transporting, mitochondrial Fo complex subunit D	Yes
ATP5L	0.008153	1.76E-05	0.539188	1.453154	ATP synthase, H+ transporting, mitochondrial Fo complex subunit G	Yes
SMPDL3A	0.014487	6.38E-05	0.538781	1.452745	sphingomyelin phosphodiesterase acid like 3A	No
TRNP1	0.042797	3.58E-04	0.538611	1.452573	TMF1-regulated nuclear protein 1	No
ANG	0.019279	1.01E-04	0.536414	1.450363	angiogenin	No
CPEB3	0.044059	3.94E-04	0.528399	1.442328	cytoplasmic polyadenylation element binding protein 3	Yes
LOC1004195	0.007851	1.40E-05	0.527237	1.441166	ring finger protein 4 pseudogene	No
ETFDH	0.008967	2.30E-05	0.52447	1.438405	electron transfer flavoprotein dehydrogenase	Yes
FUT8	0.018611	9.34E-05	0.524157	1.438093	fucosyltransferase 8	Yes
SFXN4	0.007851	1.53E-05	0.519946	1.433901	sideroflexin 4	No
FTH1	0.031399	2.42E-04	0.516888	1.430865	ferritin heavy chain 1	Yes
PPP1R13B	0.044367	4.38E-04	0.510301	1.424347	protein phosphatase 1 regulatory subunit 13B	No
DLAT	0.016639	8.17E-05	0.508035	1.422112	dihydrolipoamide S-acetyltransferase	Yes
GSTK1	0.011589	3.85E-05	0.507398	1.421484	glutathione S-transferase kappa 1	Yes
OGDH	0.016686	8.28E-05	0.506918	1.421011	oxoglutarate dehydrogenase	Yes
LDHB	0.011589	3.90E-05	0.503295	1.417447	lactate dehydrogenase B	Yes
TMEM136	0.031288	2.29E-04	0.503063	1.417219	transmembrane protein 136	No
CEMIP	0.028076	1.88E-04	0.500373	1.414579	cell migration inducing hyaluronan binding protein	No
UQCC1	0.021482	1.19E-04	0.491913	1.406308	ubiquinol-cytochrome c reductase complex assembly factor 1	No
GSTM4	0.023571	1.40E-04	0.491866	1.406262	glutathione S-transferase mu 4	No
METRNL	0.013606	5.01E-05	0.490562	1.404992	meteorin like, glial cell differentiation regulator	No
IL32	0.019193	9.96E-05	0.484049	1.398664	interleukin 32	No
PDHX	0.044059	4.01E-04	0.48243	1.397095	pyruvate dehydrogenase complex component X	Yes
ISOC2	0.011589	3.83E-05	0.478969	1.393747	isochorismatase domain containing 2	Yes
SUCLA2	0.043499	3.69E-04	0.47687	1.391721	succinate-CoA ligase ADP-forming beta subunit	Yes
ACSL4	0.044286	4.19E-04	0.473215	1.388199	acyl-CoA synthetase long-chain family member 4	Yes
COQ9	0.015085	6.98E-05	0.471526	1.386576	coenzyme Q9	Yes
CALM2	0.029122	1.98E-04	0.470024	1.385133	calmodulin 2	No
FAM162A	0.029122	2.00E-04	0.469889	1.385003	family with sequence similarity 162 member A	Yes
PRCP	0.0208	1.11E-04	0.46722	1.382443	prolylcarboxypeptidase	Yes
CTSL	0.039073	3.20E-04	0.465766	1.38105	cathepsin L	Yes
ATP5F1	0.02379	1.43E-04	0.465763	1.381047	ATP synthase, H+ transporting, mitochondrial Fo complex subunit B1	Yes
ATP5G1	0.024936	1.53E-04	0.465308	1.380612	ATP synthase, H+ transporting, mitochondrial Fo complex subunit C1 (subunit 9)	No
COX14	0.031288	2.31E-04	0.462985	1.378391	COX14, cytochrome c oxidase assembly factor	Yes
PRKRA	0.026219	1.65E-04	0.461306	1.376787	protein activator of interferon induced protein kinase EIF2AK2	Yes
ISCU	0.016	7.59E-05	0.46078	1.376285	iron-sulfur cluster assembly enzyme	Yes
COX20	0.031288	2.33E-04	0.457319	1.372988	COX20, cytochrome c oxidase assembly factor	Yes
APOO	0.044286	4.21E-04	0.448543	1.364661	apolipoprotein O	Yes
MRPL41	0.014373	6.22E-05	0.445028	1.361341	mitochondrial ribosomal protein L41	No
ESRRA	0.020372	1.08E-04	0.442483	1.358941	estrogen related receptor alpha	No

LYRM5	0.043538	3.74E-04	0.442127	1.358606	LYR motif containing 5	No
MDH1	0.023571	1.41E-04	0.44052	1.357093	malate dehydrogenase 1	Yes
SLC39A8	0.02684	1.77E-04	0.439783	1.3564	solute carrier family 39 member 8	Yes
SDHA	0.023571	1.40E-04	0.436297	1.353127	succinate dehydrogenase complex flavoprotein subunit A	Yes
UBE3B	0.0242	1.47E-04	0.431886	1.348996	ubiquitin protein ligase E3B	No
TRIM47	0.031288	2.37E-04	0.423896	1.341545	tripartite motif containing 47	No
CNBP	0.035757	2.91E-04	0.42189	1.339682	CCHC-type zinc finger nucleic acid binding protein	Yes
HADHB	0.045095	4.48E-04	0.418904	1.336911	hydroxyacyl-CoA dehydrogenase/3-ketoacyl-CoA thiolase/enoyl-CoA hydratase (trifunctional protein), bet	Yes
SOD2	0.04661	4.68E-04	0.412784	1.331253	superoxide dismutase 2, mitochondrial	Yes
NACA	0.029122	2.01E-04	0.408295	1.327117	nascent polypeptide-associated complex alpha subunit	Yes
WWP1	0.047892	4.89E-04	0.404271	1.32342	WW domain containing E3 ubiquitin protein ligase 1	No
UQCRC1	0.047892	4.88E-04	0.402235	1.321554	ubiquinol-cytochrome c reductase core protein I	Yes
CS	0.044052	3.88E-04	0.400584	1.320042	citrate synthase	Yes
ANTXR1	0.035188	2.84E-04	0.399578	1.319122	anthrax toxin receptor 1	No
COQ3	0.033704	2.69E-04	0.399078	1.318665	coenzyme Q3, methyltransferase	Yes
SLC25A5	0.044059	4.00E-04	0.395352	1.315264	solute carrier family 25 member 5	Yes
NDRG3	0.04014	3.34E-04	0.395323	1.315237	NDRG family member 3	Yes
HSD17B12	0.032132	2.54E-04	0.394497	1.314484	hydroxysteroid 17-beta dehydrogenase 12	Yes
SMARCA5	0.047913	4.92E-04	0.390085	1.310471	SWI/SNF related, matrix associated, actin dependent regulator of chromatin, subfamily a, member 5	Yes
RPL9	0.044286	4.29E-04	0.386409	1.307136	ribosomal protein L9	Yes
AMY1A	0.044321	4.35E-04	0.377776	1.299337	amylase, alpha 1A (salivary)	No
LAMB2	0.044286	4.24E-04	0.3763	1.298009	laminin subunit beta 2	Yes
DNAJC4	0.044286	4.31E-04	0.370727	1.293004	DnaJ heat shock protein family (Hsp40) member C4	No
GON7	0.044286	4.18E-04	0.370591	1.292882	GON7, KEOPS complex subunit homolog	No
CHCHD10	0.047455	4.79E-04	0.360415	1.283795	coiled-coil-helix-coiled-coil-helix domain containing 10	No
NDUFV1	0.043538	3.78E-04	0.360207	1.28361	NADH:ubiquinone oxidoreductase core subunit V1	Yes
AP5M1	0.049254	5.14E-04	0.351922	1.276259	adaptor related protein complex 5 mu 1 subunit	No
ACAT1	0.04661	4.66E-04	0.351494	1.275881	acetyl-CoA acetyltransferase 1	Yes
CIPC	0.049868	5.29E-04	0.342753	1.268174	CLOCK interacting pacemaker	No
COX5B	0.048584	5.04E-04	0.342519	1.267968	cytochrome c oxidase subunit 5B	Yes

Table S5. Correlation of cell cycle genes with *PPARGC1A* expression in human PCa. Related to Figure 5 and Figure S5.

Gene B	Gene A	TCGA Correlation	TCGA.Correlation p value	TCGA Adjusted p value		
CCNB1	PPARGC1A	-0.465150553	6.08E-28	7.90E-27	Adjusted p-value <1e-06; R < -0.3	#, *
NUF2	PPARGC1A	-0.463749346	9.17453E-28	1.37618E-26		#, *
GTSE1	PPARGC1A	-0.45853943	4.1758E-27	4.1758E-26		#, *
MELK	PPARGC1A	-0.44013991	7.18591E-25	3.59295E-24		#
TACC3	PPARGC1A	-0.42941351	1.25397E-23	9.40476E-23		#, *
EME1	PPARGC1A	-0.416131135	3.76447E-22	2.44691E-21		#, *
KIF2C	PPARGC1A	-0.413006471	8.20008E-22	2.73336E-21		#, *
ERCC6L	PPARGC1A	-0.409146127	2.12159E-21	9.19355E-21		#
NUSAP1	PPARGC1A	-0.398165414	2.96479E-20	1.48239E-19		#, *
CDC6	PPARGC1A	-0.395338483	5.75462E-20	1.87025E-19		#
RAD54L	PPARGC1A	-0.383605174	8.4374E-19	3.16403E-18		#, *
KIF14	PPARGC1A	-0.374831983	7.37741E-18	1.84435E-17		#, *
SKA1	PPARGC1A	-0.368702779	2.19314E-17	6.57943E-17		#, *
SHCBP1	PPARGC1A	-0.357644829	2.21024E-16	5.52559E-16		#, *
CENPM	PPARGC1A	-0.348356345	1.43714E-15	3.73655E-15		#
CCNE2	PPARGC1A	-0.345353587	2.59798E-15	5.62895E-15		#
CDT1	PPARGC1A	-0.341545855	5.45438E-15	1.01296E-14		#, *
NCAPG2	PPARGC1A	-0.335054469	1.88697E-14	4.04351E-14		#
ZWILCH	PPARGC1A	-0.334284794	2.18192E-14	4.0911E-14		#
KIF11	PPARGC1A	-0.33311297	2.71974E-14	5.43948E-14		#, *
KIF15	PPARGC1A	-0.316886213	5.22444E-13	8.70741E-13		#
RAD51AP1	PPARGC1A	-0.298071968	1.29321E-11	1.93982E-11	Adjusted p-value <1e-06; R < -0.2	#
KIF23	PPARGC1A	-0.289216887	5.41367E-11	7.73381E-11		#
ORC1	PPARGC1A	-0.279747098	2.37087E-10	3.23301E-10		#, *
CENPI	PPARGC1A	-0.265765007	4.18147E-09	6.79489E-09		#
DDIAS	PPARGC1A	-0.258603309	5.26344E-09	7.60274E-09		#
ESCO2	PPARGC1A	-0.25607847	7.75227E-09	1.00779E-08		#
E2F8	PPARGC1A	-0.173349083	0.000111278	0.000131511	Adjusted p-value <0.05; R < -0.1	#
PRR11	PPARGC1A	-0.169666583	0.00024489	0.000306113		#
MASTL	PPARGC1A	-0.165224052	0.000222397	0.000247108		#
WDHD1	PPARGC1A	-0.153015236	0.000635708	0.000733509		#
POLE	PPARGC1A	-0.098358298	0.028661627	0.030708886	NS	
INCENP	PPARGC1A	-0.065775304	0.143935559	0.143935559		#
CEP170	PPARGC1A	-0.006687397	0.882018663	0.882018663		
BARD1	PPARGC1A	0.063333987	0.162874499	0.176447374		
ST18	PPARGC1A	0.094628586	0.035875551	0.035875551		
KIF26B	PPARGC1A	0.265263579	2.04062E-09	2.55077E-09		
PLK2	PPARGC1A	0.310016497	1.73173E-12	2.88621E-12		

#Genes significantly (p<0.05) correlated with DFS listed in Table S4
*Top 14 genes significantly correlated with DFS listed in Table S4

Table S7. PCR primers used in this work. Related to Figures 5, 6 and S6.

Gene	Forward Primer (5' to 3')	Reverse Primer (5' to 3')	SOURCE	IDENTIFIER
CCNB1	AGAGCATCTAAGATTGGAGAG	CCATGTCATAGTCCAACATAG	Sigma-Aldrich	N/A
CDT1	CCTGGGGAAATGGAGAAG	TTGTCCAGCTTGACGTAG	Sigma-Aldrich	N/A
GTSE	CCTCAGGCACTTAACTTTTC	ATGAGAGGAAGGTCAATGAG	Sigma-Aldrich	N/A
KIF2C	CAGTAGTTTTCCAAACTGGG	TAGGATCAGTCATAGTAAGTGG	Sigma-Aldrich	N/A
KIF11	GGAAACTCTGAGTACATTGG	GAGTTTCTGATTCACTTCAGG	Sigma-Aldrich	N/A
KIF14	AAGACAGAATTTGTGGAAGG	TAGTCGATCTCCATTAGTGTG	Sigma-Aldrich	N/A
NUF2	CAATATAAATCCTCTGCGGAC	GAATCAAGTCTCTCCAGTTT	Sigma-Aldrich	N/A
NUSAP1	AGACTCCAGTCTCCAATAAG	TTTAGCTTTCCTTTGTGTGG	Sigma-Aldrich	N/A
ORC1	AACTTAGGTAACCCTCAGATG	CTGAGATCTCTTAGAAGGTGAG	Sigma-Aldrich	N/A
RAD54L	AAAAAGATGCCTTGGTTCTG	CTGAGAATAGGGTCAACAAC	Sigma-Aldrich	N/A
SHCBP1	GAGGTATGTGTTTGGTTATCAG	GCAGACAATGGATCACTATG	Sigma-Aldrich	N/A
SKA1	GATCTGGAACAATTATGCTCTC	CTGGCCACAGTTTCTTAATG	Sigma-Aldrich	N/A
TACC3	AAGACTAAAGAGAACGAGGAG	GAACATGACACCTAAGAGAATC	Sigma-Aldrich	N/A
UBC	CGTCACTTGACAATGCAG	TGTTTTCCAGCAAAGATCAG	Sigma-Aldrich	N/A
